# *Sida* L.: Ethnobotany, Pharmacology, and Phytochemistry: A Review

**DOI:** 10.3390/plants14193115

**Published:** 2025-10-09

**Authors:** Enrique Jiménez-Ferrer, Maribel Herrera-Ruiz, Yrvinn Campos-Vidal, Gabriel Flores-Franco, Nayeli Monterrosas-Brisson

**Affiliations:** 1 Centro de Investigación Biomédica del Sur, Instituto Mexicano del Seguro Social (IMSS), Xochitepec 62740, Mexico; enriqueferrer_mx@yahoo.com (E.J.-F.); yrvinncv@gmail.com (Y.C.-V.); 2 Centro de Investigación en Biodiversidad y Conservación, Universidad Autónoma del Estado de Morelos (UAEM), Cuernavaca 62209, Mexico; gabrielflores@uaem.mx; 3 Facultad de Ciencias Biológicas, Universidad Autónoma del Estado de Morelos (UAEM), Cuernavaca 62209, Mexico

**Keywords:** *Sida* spp., traditional medicine, ethnobotany, pharmacology, alkaloids, secondary metabolites

## Abstract

The genus *Sida* includes about 200 species worldwide. Its history in folk medicine is extensive, as it has been used to treat various conditions such as inflammation, pain, and nervousness. Pharmacologically, preclinical studies have attributed antioxidant, anti-inflammatory, analgesic, and sedative properties to *Sida*, related to the presence of alkaloids, flavonoids, and coumarins. A relevant point of this review is that, despite the number of *Sida* species, only 17 of them have pharmacological reports, emphasizing their great potential, such as *S. rhombifolia*, which requires further research to conduct clinical trials, since only *S. cordifolia* has undergone clinical trials with arthritis patients. The objective of this review was to conduct a literature search across different databases, to update the available information on the ethnomedical use, phytochemistry, and pharmacological potential of 17 *Sida* species. The information compiled in this review aims to highlight the therapeutic importance of *Sida* species, whether they have been researched or not. This genus is a promising field for scientific advances in chemistry and pharmacology. It also has the potential to create appropriate conditions for obtaining plant material in a systematic and sustainable manner, to prevent overexploitation and encourage clinical studies leading to pharmaceutical formulations for therapy.

## 1. Introduction

Traditional medicine consists of a wealth of knowledge and skills derived from the theories, beliefs, and experiences of various indigenous cultures. This knowledge, whether or not it can be explained by modern science, is applied to promote well-being, prevent disease, make diagnoses, and offer treatments for physical or mental conditions [1]. Medicinal use of plants dates back to prehistoric times [2]. Archaeology, despite being an inexact science, uses partial remains to reconstruct the past. Archaeological findings, such as bones showing signs of survival from trauma or disease, suggest that prehistoric hominids and Neanderthals used medicinal and antibacterial plants to heal themselves and recover from injuries [3]. Since early ages, humans have faced a great challenge in searching for sustenance, shelter, and medicines in nature. Traditional medicine is one of the oldest forms of healing in the world, as it has been practiced since ancient times. Many indigenous communities around the world continue to try different traditional remedies, and their knowledge is passed on by word of mouth from generation to generation [4].

In Mexico, traditional medicine dates to pre-Hispanic times and remains in practice today, contributing to the treatment of ailments among Mexicans. Evidence of its use is recorded in the Florentine Codex, created by the Franciscan missionary Fray Bernardino de Sahagún, along with indigenous elders and artists, to repaint and rewrite their history. It shows the complex and sophisticated medical system practiced in Mesoamerica before the Spanish conquest [5], as well as in the Cruz-Badiano Codex, also known as *Libellus de Medicinalibus Indorum Herbis*, written in Nahuatl by an indigenous man named Martín de la Cruz and translated into Spanish by Juan Badiano. It is of great importance in Mexico because it is the first treatise on Mexica herbal medicine, thus considered a valuable testimony to pre-Hispanic medical knowledge [4].

As a megadiverse country, Mexico combines one of the most abundant floras on the planet with vast cultural wealth, positioning itself as one of the key points of biocultural diversity worldwide [6]. With approximately 70 Indigenous Peoples and more than 60 dialects, Mexico’s cultural diversity is significant. Because of its biological megadiversity, a wide variety of traditions related to the use of plants has developed, and consequently, there is a wealth of ethnobotanical knowledge [7].

It should be noted that Mexico has about 30,000 species of medicinal plants, and many of them are endemic. However, only around a thousand have been investigated, revealing the enormous potential of species that remain to be explored [6].

According to the World Health Organization (WHO), around 80% of the world’s population relies primarily on medicinal herbs to satisfy their basic health needs [8]. In Mexico, they are the only medical alternative for approximately 40 million Mexicans who do not have access to healthcare services [9,10].

Furthermore, plant-based product development and extracts used in traditional medicine are limited because of the lack of scientific evidence and poor practices when developing herbal medicines. Therefore, it is important to encourage pharmacological, phytochemical, and toxicological research on plant species potentially useful for treating various diseases [11]. Today, it is known that pharmaceutical companies often use plants, since several drugs with therapeutic purposes are based mainly on active ingredients derived from traditional medicinal plants [12]. In fact, these plants are considered living chemical factories where a variety of secondary metabolites are synthesized, which exert different biological activities with the potential to improve human health [13], and new therapies based on natural resources have been researched for years, leading to several important discoveries, such as antibiotics, anticancer agents, anti-inflammatory, and analgesic compounds [14].

### 1.1. Malvaceae Family

The Malvaceae family is commonly known as the hibiscus, mallow, or cotton family. It comprises 244 genera and 4225 species of flowering plants [15,16]. It includes some species used in traditional medicine to relieve kidney problems, acute dysentery, body burning, urinary incontinence, seminal weakness, and hemorrhoids [2]. Several bioactive compounds isolated from various Malvaceae species (carotenoids, phenolic acids, flavonoids, coumarins, alkaloids, lignans, cardiac glycosides, sterols, terpenes, and polysaccharides) have been reported to exert cytotoxic, anticarcinogenic, antioxidant, and anti-inflammatory effects, among others [14,17].

### 1.2. Genus Sida *L.*

*Sida* L. was described by Carl Linnaeus and published in *Species Plantarum* [18]. It is one of the most diverse genera belonging to the Malvaceae family, with around 200 species found in tropical and subtropical regions around the world, as well as being of great ethnomedical importance [19]. Of these, 189 species are found in North and South America, 35 have been recorded in Mexico [20] and 10 in Morelos, Mexico [21]. They can be found as weeds in grasslands and wastelands in tropical and subtropical regions around the world [19]. Some of them are classified as invasive plants or weeds and can infest crops, thereby affecting agriculture [22]. However, some *Sida* plants have ethnomedical importance and are used to treat various disorders [17].

### 1.3. Botanical Description

They are annual or perennial herbs, subshrubs, or shrubs 0.5–2.0 m tall with star-shaped hairs, simple and/or granular, often woody with strong and deep roots. Leaves are petiolate, with stipules; simple blades with margins that never appear entire and without foliar nectarines. Flowers usually appear in summer, are actinomorphic, axillary, solitary, or with accessory nirnules; calyx campanulate, mostly deeply pentaparted, sometimes accrescent; possessing five asymmetrical petals, barely welded together at the base by the staminal tube, cream, yellow, pink, or purplish, often with a reddish spot at the base; androecium monadelphous, with numerous stamens; ovary carpellate (5–15), with free stigmatic branches (5–15); carpels muticulate or with two vertical aristae, dehiscent between the aristae. Pendulous seeds. Fruits are 5-carpellate with slender mericarps and relatively large calyxes enclosing and concealing the fruits [23].

### 1.4. Uses

It has been reported for a long time that the fiber of these species is comparable in quality to jute in Europe and Asia. That is why *S. rhombifolia* and *S. cordifolia* have been cultivated in India for centuries. Their fibers have a high percentage of cellulose and are useful in paper, rope, and broom manufacturing [24]. Currently, species of this genus have been considered weeds with no practical application outside of traditional medicine [24], since it has significant ethnomedical importance. Throughout history, various parts of these plants have been widely used in traditional indigenous medicine to treat a wide range of ailments, including neurological and uterine disorders, headaches, tuberculosis, diabetes, malaria, ulcers, wounds, and rheumatic and heart problems [25].

Approximately 140 chemical compounds have been identified in this genus, the most active ones being alkaloids and flavonoids, as they are involved in most of the pharmacological and biological properties of the genus [26]. Pharmacological reports available to date support various ethnomedical uses of the genus *Sida*, confirming a range of biological activities such as anti-inflammatory, anti-rheumatic, antipyretic, antitumor, anti-HIV, hepatoprotective, antimicrobial, and immunostimulant effects [27].

Sierra de Huautla Biosphere Reserve (REBIOSH) is one of 48 reserves recognized by CONANP (Comisión Nacional de Áreas Naturales Protegidas). It covers an area of 5903 km^2^ where 22 genera belonging to the Malvaceae family have been identified, including *Sida*. Some floristic records list eight species of this genus in the REBIOSH, among them *S. abutilifolia* Mill., *S. acuta* Burm.f., *S. ciliaris* L., *S. glabra* Mill., *S. jamaicensis* L., *S. rhombifolia* L., *S. spinosa* L., *S. viarum* A. St. Hil. [21], and *S. neomexicana* A. Gray [28].

REBIOSH is managed by CONANP and the Centro de Investigación en Biodiversidad y Conservación at the Universidad Autónoma del Estado de Morelos (UAEM). Some of our research group members are affiliated with UAEM and collaborate with colleagues from the Centro de Investigación Biomédica del Sur of the Instituto Mexicano del Seguro Social (CIBIS-IMSS). The objectives of the reserve are to preserve biodiversity while promoting the economic and social development of the area through sustainable projects [29].

Therefore, our group proposed a research project to investigate the pharmacological and chemical properties of *Sida rhombifolia* to validate some of its ethnopharmacological uses, as well as to encourage moderate harvesting of this natural resource and promote sustainable cultivation techniques that could benefit REBIOSH’s surrounding communities and eventually develop a phytomedicine.

To this end, the objective of this review was to conduct a literature search concerning the ethnomedical, phytochemical, and pharmacological potential of the genus *Sida*, in order to update the information provided by [14,23], but mainly to emphasize its importance in the therapeutic field and, therefore, to guide further research on these plants through experimental designs and compare the results obtained with current references, providing a scientific chemical-pharmacological basis for the future design of phytomedicines.

Despite the worldwide distribution of *Sida* species and the extensive knowledge of their medicinal use in various cultures, there is a notable scarcity of rigorous chemical-pharmacological studies that validate and explore them in greater depth. This discrepancy represents a research opportunity, as ethnobotanical evidence can serve as a promising starting point for the development of phytomedicines.

Therefore, considering the ethnomedical use of *Sida* species, the question we posed was: Is there enough chemical-pharmacological and preclinical research on these plants to justify and provide a basis for clinical trials in humans?

## 2. Materials and Methods

### 2.1. Bibliographic Search

An extensive search of various articles, books, and digital libraries was conducted between 20 September 2024, and 30 July 2025. We consulted databases such as PubMed, Google Scholar, Scopus, SciELO, and ScienceDirect. The search criteria included terms related to the Malvaceae family, the genus *Sida*, and its ethnomedicinal uses, pharmacological potential, and phytochemistry. Specific terms included: “family Malvaceae”, “*Sida* gen us”, “traditional use of *Sida* genus plants”, “medicinal uses of *Sida* genus”, “plants to treat vascular disorders”, “plants to treat hypertension”, “plants to treat psychiatric disorders”, “ethnopharmacological studies of *Sida* genus”, “biological activities of plants of *Sida* genus”, “pharmacology of *Sida* genus”, “*Sida* genus anti-inflammatory activity”, “*Sida* genus antioxidant activity”, “*Sida* genus antihypertensive activity”, “*Sida* genus anxiolytic activity”, “*Sida* genus antimicrobial activity”, “*Sida* genus antidepressant activity”, “pharmacological studies of *Sida* genus”, and “*Sida* genus phytochemistry”. Other sources used in this study included books, book chapters, and digital libraries (https://herbanwmex.net/portal/index.php, http://www.medicinatradicionalmexicana.unam.mx/, and https://www.biodiversidad.gob.mx/diversidad/medicinal, revised in 7 June 2025).

Names as well as scientific synonymy of plants were verified in https://powo.science.kew.org/ (accessed on 30 January 2025), https://identify.plantnet.org/es (accessed on 30 January 2025), https://enciclovida.mx/ (accessed on 30 January 2025), and https://mexico.inaturalist.org/ (accessed on 30 January 2025).

The search resulted in a master list of 17 *Sida* species, including the available data on scientific synonymy, their traditional uses, phytochemistry, as well as the pharmacological activities of each listed species. Eligible articles were evaluated for inclusion in the study using inclusion/exclusion criteria established by the authors.

### 2.2. Inclusion and Exclusion Criteria

Full-text articles that included ethnopharmacological or pharmacological data with descriptions of trials and significant results of the chosen *Sida* species, published in peer-reviewed journals, reports, books, theses, and dissertations up to 30 July 2025, were included.

**Figure 1:**
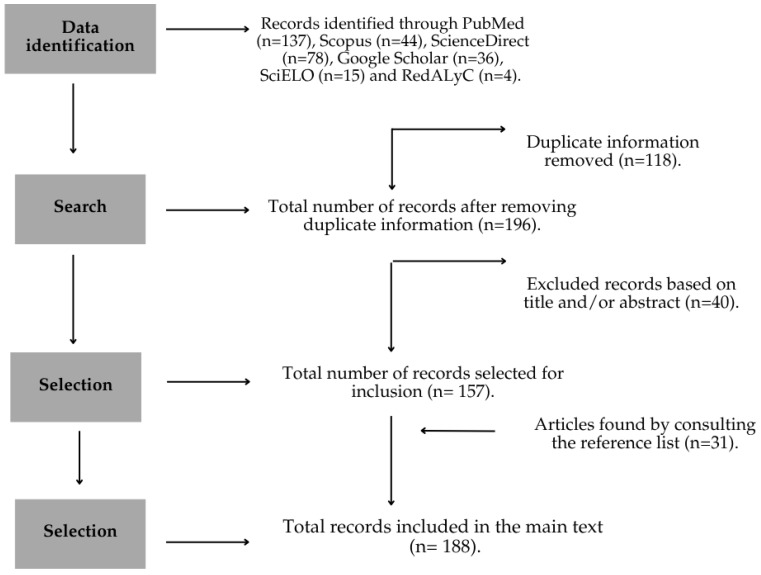
Data selection methods applied in this review.

Inclusion criteria took into account any year of publication and no geographical restrictions; however, preference was given to those with a publication date after [14,23]. Documents written in English were included, as well as those in other languages only when they provided essential information unavailable in other texts, websites, or media (Figure 1).

### 2.3. Data Extraction

Information available regarding different aspects of the *Sida* species included in the study was compiled in a database created using Microsoft 360 Copilot Excel (Microsoft Corporation, Redmon, WA, USA). Three authors extracted relevant data from the included documents regarding the botanical, ethnobotanical, pharmacological, phytochemical, and bioactive descriptions of the natural products reported for some *Sida* species. Pharmacological data, such as the model employed, biological activity reported, statistical significance, type of extract (solvent and plant part plant used), and dose administered were also included, if reported. Other authors reviewed the comprehensiveness and treatment of the collected data.

### 2.4. Data Presentation

Compiled data containing information on botanical description, ethnobotany, biological activities, methodology used, type of extract, and doses employed, were synthesized and integrated into Table 1 and Table 2. Data referring to the chemical compounds found in these plants were summarized in Table 3. “N/R” was written when the information was not reported.

## 3. Results

For thousands of years, plants have been a central element of traditional medicine, with different parts such as fruits, leaves, and roots used to treat various diseases [30].

### 3.1. Botanical Description of Species from the Genus Sida 

#### 3.1.1. *Sida acuta* Burm.f.

Erect shrubs or subshrubs, growing straight and reaching up to 1.5 m in height. Distichous branching, glabrescent stems. Leaves are elongated and alternate, green on both sides, petiolate; blades lanceolate to ovate, with serrated margins, with falcate stipules. Solitary or paired axillary flowers, hermaphrodite, actinomorphic, and pentamerous; calyx with solitary sepals often ciliate, about half-divided, basally 10-costate; corolla with white, yellow, or yellow-orange petals fused at base. Androecium Monadelphous; filaments attached to the base of the corolla. The fruits are green, and when dry they turn brown, with seeds bearing short hair. This plant is pantropical, found in tropical and subtropical regions around the world. It is native to Central America, but has spread throughout tropical and subtropical areas, including Africa, Asia, and the Pacific Islands, and it inhabits warm and semi-warm climates from 100 to 1200 m above sea level. It usually grows along roadsides, associated with tropical deciduous, sub-deciduous, evergreen, and induced grasslands. The flowering period occurs throughout the year in warm climates, intensifying during the rainy season 2*n* = 28 [31,32,33].

#### 3.1.2. *Sida ciliaris* L.

Perennial herbs, branched from the base. Stems procumbent, scabrous with stellate trichomes, adpressed. Leaves with a petiole 2–7 mm long; linear to obovate-lanceolate, stipules, 3–6 mm long, partially canescent on the petiole; lamina linear to ovate-lanceolate, ovate, or obovate, 6–18 × 2–8 mm, acute, obtuse, or closely truncate, with rounded or closely truncate base and dentate margin in the middle or in the distal one-third, upper side glabrous or subglabrous, underside scabriform with adpressed stellate trichomes. Axillary inflorescences uniflorous, clustered near the apex of branches, forming a capituliform synflorescence; pedicel approximately ≤2.5 mm in length, puberulent; calyx about 3–4.5 mm long, ca. ⅔ split, hirsute and ciliate; lobes triangular, acute or acuminate. Petals 6–8 × 3–5 mm, orange-pink with a dark red basal blotch. Staminal column 1–2 mm long; filaments yellow or pink; yellow anthers. Style with 5–7 yellow or red branches 1.5–2 mm long; yellow or dark red stigmas. Schizocarpic fruit depressed, 2–2.5 × 4–5 mm; mericarps 5–7, subinermous, tuberculate or muricate on dorsum, laterally reticulate, puberulous with minute stellate trichomes at the tubercle apex. Seeds closely reniform, about 1–1.5 mm long, brown, puberulent. This plant is widely distributed, ranging from the southern United States to South America, including Central America and the Antilles. In Mexico, it is found in the center, the Gulf, northeast, northwest, southeast, and southwest of the country. It can be found in habitats such as grasslands, human-altered land such as yards and roadsides, and abandoned fields. It flowers throughout the year. Chromosome number 2*n* = 16 [33,34,35].

#### 3.1.3. *Sida cordata* (Burm f.) Borss. Waalk.

Prostrate or semiprostrate herbs, up to 80 cm tall. Profusely branched at the base, stellate-pubescent on all sides, mixed with simple spreading hairs on branches, petioles, pedicels, and calyx. Leaves 1–5.5 cm in length, 1–5 cm in width, narrowly to widely ovate, lanceolate on terminal branches, crenulate to serrate, cordate or rounded at base, acuminate at apex, lower surface stellate pubescent, upper surface with stellate and simple, stellate hairs, strigose; filiform stipules, about 2–4 mm long; petiole 1–4 cm in length. Axillary flowers, solitary or forming a pseudoraceme due to leaf-reduction on terminal parts; pedicel 1.5–2.5 cm in length, in fruit up to 3.5 cm, articulated slightly above the middle; calyx fused to the middle, up to 4–5 mm long and wide; lobes ca. 2 mm wide, acuminate; pale yellow corolla, measuring 7–8 mm wide. Staminal column 2–3 mm in length, simply pilose. Depressed globose fruit, 3–4 mm in diameter, pubescent on top, beaked; mericarps 5, dehiscent, membranous, smooth, disintegrated on radial surfaces, 2.5 mm long. Brown seeds, about 2 mm long, glabrous. This is a highly variable species in indumentum and inflorescence. Variation in inflorescence sometimes gives the illusion of the presence of at least two taxa. Continuous variation in inflorescence has been observed in herbarium specimens, so it is not advisable to separate taxa based on this character. This pantropical species is native to Asia—specifically India, Sri Lanka, and parts of China—and Africa. It has also been introduced to other regions, including the southeastern U.S., the Caribbean, and parts of South America. Chromosome number 2*n* = 16 [14,35].

#### 3.1.4. *Sida cordifolia* L.

Erect shrubs or subshrubs to 1.5 m tall, with ascending branches, a tomentose stem having stellate trichomes. Leaf blades to 6 cm long, ovate-cordate, silky, with dentated-serrated margin. Flowers in axillary cymes, rarely alone; calyx 6–7 mm long, prominently 10-ribbed, tomentose; corolla with yellow-orange petals. Schizocarp fruit with 10 reticulate mericarps laterally and with two prolonged edges in the distal portion, pubescent with retrorse trichomes. Seeds are reniform and glabrous. It grows in semi-shaded places in wet, sandy soils. It is widely distributed over tropical and subtropical regions, with a notable presence in Asia, Africa, and the Americas. In some areas, it is considered an invasive weed, and in others it is used for medicinal purposes. It flowers all year round. Chromosome number 2*n* = 28 [34,35].

#### 3.1.5. *Sida cordifolia* L. subsp. *maculata* (Cav.) Marais

A perennial herbaceous plant or shrub up to 1 m tall, with ovate, heart-shaped, green to gray-green leaves and coarsely toothed margins. Its flowers are bright yellow and grouped in clusters at the ends of the stems and branches. The fruits are beak-shaped mericarps with 3–4 mm long edges. These plants are commonly found as weeds in cultivated and disturbed areas. It occurs in tropical and subtropical areas, including eastern, western, central, and southern Africa, as well as eastern, southern, and southeastern Asia, and parts of North and South America. Flowering time: throughout the year. *S. cordifolia* is known to have a chromosome number of 2*n* = 28. This subspecies, *maculata*, falls under that count [36].

#### 3.1.6. *Sida glutinosa* Comm. ex Cav.

Suffrutescent herbs to 1.5 m tall, branched. Erect stems, vesiculose with numerous glandular trichomes, long simple trichomes, patent, occasionally stellate. Leaves petiolate, 1–5.5 cm long; stipules subulate, 2–3 mm long; leaf blades ovate to ovate-lanceolate, 4–9.5 × 3–7 cm (commonly smaller in inflorescence leaves), acuminate, cordate at base, and with serrated margins, pubescent to scabriuscule (winding) on the upper side and pubescent to tomentulous on the underside by stellate trichomes, denser on this side. Terminal inflorescences, in panicles, rarely axillary and uniflorous; pedicel 0.4–2 cm long, articulated in the distal half, puberulous to pubescent; calyx 3.5–4 mm long and lobed by ¼–⅓, pubescent; lobes triangular, closely acute or apiculate; petals 5–6 × 4–5 mm, pale orange-yellow. Staminal column 1–1.5 mm long; yellow filaments and anthers. Style with five yellow or reddish branches ca. 1.5 mm long; yellow or red stigmas. Schizocarp conical, 4–4.5 × 3.5–4 mm (spines included); mericarps 5, with two subapical spines ca. 2 mm long, dorsally and laterally reticulate, puberulous mostly towards the apex and on spines by simple trichomes, antrorse. Seed ± reniform, ca. 2 mm long, brownish, smooth. This species is native to a wide area from southwestern Mexico to Ecuador, the Caribbean, and from central to eastern Brazil and Paraguay, found in deciduous forests, savannas, and disturbed areas, often in shade, in the north-central and Pacific regions; 0–1100 m; flowers and fruits throughout the year. Chromosome number: 2*n* = 32 [37,38].

#### 3.1.7. *Sida hyssopifolia* C. Presl

Erect shrub, 0.5–1.5 m; stems stellate-pubescent; stipules narrowly falcate, uninervate. Leaves whorled, 1–6 × 0.5–3 cm, elliptic to subrounded (sometimes narrowly), acute to rounded at base, acute to rounded at apex, distally serrate, pubescent on the upper side with trichomes usually adpressed, simple. Inflorescences axillary, solitary, paired, or several grouped distally; pedicels 1–6(−18) mm. Flowers with calyx 5–6(−10) mm; petals orange-yellow with a red base. Fruits 0.35–0.4 cm diameter, minutely papillose to simple-pubescent distally; mericarps 6–9, cinnamon to dark gray, laterally reticulate or nearly smooth, obtuse. It is distributed from northern Mexico to Costa Rica and in some regions of Africa; in pine-oak forests, pastures, and adjacent roads, in the north-central and Pacific regions, between 0 and 1000 m above sea level. It flowers from September to January. Chromosome number: not reported [38].

#### 3.1.8. *Sida javensis* Cav.

Prostrate herbs. Stems branched, 0.2–1 m long, rooting at the nodes, ±glabrous, pubescent, or rarely pilose. Leaves rounded-ovate, 0.5–5 × 0.5–5.5 cm, unlobed to very shallowly three-lobed, acute at the apex, cordate at base, distinctly serrate or biserrate, appressed-pilose; petiole 0.5–5 cm long; stipules linear to lanceolate, 2–2.5 mm long. Flowers solitary; pedicel 0.5–2.5 cm long, jointed above the middle; calyx 5–7 mm long with ribbed tube, lobes 2–5 × 1.5–4 mm, acute, three-veined; corolla yellow or orange-yellow; petals 6–8 mm long. Staminal column 1.5–3 mm long, hairy. Mericarps 5, 2.5 mm long, strigose (stems, leaves, or other organs covered with stiff or spiny hairs), back with central ribs, sides thin-walled, partly disintegrating, lateral edges prominent; beak of two awns 0.5–1.5 mm long. The distribution of this species is widespread but somewhat irregular. It is native to Asia, including Indonesia (Java, specifically), Malaysia, the Philippines, and Taiwan. However, it can also be found in India, Indochina, and parts of Africa. Chromosome number: 2*n* = 26 [38,39,40].

#### 3.1.9. *Sida linifolia* Juss. ex Cav.

Erect to ascending subshrubs to 1.5 m tall. Pubescent to hirtulate stems with 2–5-radiate, simple long, patent trichomes, longitudinal lines of small stellate and other spaced catenulate trichomes; petioles to 7 mm long, usually shorter. Leaf blades linear to lanceolate (2–11.5 × 0.2–1 cm), acute base, rounded or subtruncate and margin entire, ciliate, hirtulate on both faces by long and a few 2(-3)-radiate simple trichomes. Terminal corymbose inflorescences; pedicel 4–11 cm long, articulated in the distal third part, pubescent; calyx 4–6 mm long, ca. ½ lobed, hirsute and ciliate; lobes triangular, acute to acuminate; petals about 7–10 × 5–7 mm, white becoming yellowish, with dark red basal blotch. Staminal columns usually 2–3 mm long; filaments pale yellow; anthers yellow. Style with 6–8 red branches ca. 1 mm long; stigmas red. Depressed schizocarp, 2.5–3 × 4–5 mm; mericarps 6–8, inert, smooth to weakly reticulate-costate, subglabrous but with a few minute glandular trichomes. Seeds reniform, somewhat angular, about 1.5–2 mm long, brownish, smooth. The native range of this species is Tropical and Subtropical America, and Tropical Africa; it is found in dry savannas, brushy and grassy slopes, and fields. Flowers commonly during the rainy season. Chromosome number: not reported [32,34].

#### 3.1.10. *Sida mysorensis* Wight & Arn.

Subshrubby or erect herbs, up to 1 m tall. Stem with minute stellate hairs, multicellular glandular hairs, and long simple hairs; stipules filiform, about 5 mm; petiole ~1–3 cm, pilose; leaf blades ovate-cordate, about 3–6 × 2.5–4.5 cm, viscid-stellate on both sides with minute stellate hairs and multicellular glandular hairs, cordate base, margin crenulate, apex acuminate. Solitary or paired flowers, axillary or subterminal, often on reduced, congested axillary buds; pedicel slender, about 2–6(–15) mm, articulated at or above the middle section; calyx broadly campanulate, usually 6–8 mm, sparsely pilose with long hairs, lobes 2.5–3 × ca. 2.5 mm, acute or acuminate; yellow corolla, 1–1.2 cm in diameter; obtriangular, glabrous petals; staminal column present. Schizocarp fruit, nearly globose, about 3–4 mm in diameter; mericarps 5, ovoid-tetrahedral, about 2.5 mm, smooth, apex sparsely hairy, not very acute, enclosed in a persistent calyx. Ovoid seeds, slightly trilobed, about 2 mm, glabrous. It is distributed across Asia, from south to southeast, in tropical and subtropical regions, and grows in grassy slopes, roadsides, and forest boundaries. Chromosome number: not reported [40,41].

#### 3.1.11. *Sida planicaulis* Cav.

Herbs or subshrubs, perennial, branches distichous, planar, 0.3–1 m. Stems erect, with simple 1–2 mm hairs, sometimes also minutely stellate-hairy. Leaves distichous; stipules free from petiole, subfalcate, 4–10 × 0.5–1 mm or less, often exceeding petiole, margins ciliate; petiole 5–6 mm, 1/15–1/5 blade length, hirsute; blade elliptic-lanceolate, 2.5–9 × 1–4 cm, two times longer than wide, base rounded, margins short-serrate at least distally, entire basally, apex acute, surfaces glabrous or with minute scattered stellate hairs and simple appressed antrorse hairs. Inflorescences axillary congested glomerules, sometimes solitary or paired flowers. Pedicels 0.3–0.5 cm, subequal to or shorter than the subtending petiole, shorter than to subequal to the calyx. Flowers: calyx ribbed, 5–6 mm, often ciliate, with scattered minute stellate hairs, lobes triangular; petals yellow, 8–10 mm; staminal column glabrous; style 7- or 8-branched. Schizocarps subconic, 6–7 mm diam., glabrous or axially minutely puberulent; mericarps 7 or 8, 3 mm, smooth dorsally, laterally somewhat reticulate, apex spined, spines 2 mm, apically minutely puberulent. It is found in Brazil, where it is native, and in the southeastern United States, particularly in Florida. Chromosome number: 2*n* = 28 [22].

#### 3.1.12. *Sida rhombifolia* L.

Erect subshrubs or prostrate plants, many-branched, up to ca. 1 m tall. Stems finely stellate-puberulent, mealy; petiole up to 2–5(−8) mm, stellate puberulent; spiny stipules, ca. 3–5 mm. Leaves rhombic to oblong-lanceolate or obovate, rarely linear-lanceolate, up to 1–4.5 × 0.6–2 cm, abaxially stellate pilose, grayish white, adaxially sparsely stellate pilose to subglabrous, broadly cuneate base, margin dentate, apex obtuse to acute. Solitary flowers, axillary; pedicel 1–2.5 cm, densely stellate tomentose, articulated above the middle; calyx cup-shaped, about 4–5 mm, abaxially stellate pubescent, triangular lobes, acute apices; corolla ca. 1 cm in diameter; yellow petals, obovate, ca. 8 mm, attenuated base, apex rounded; filamentous tube 4–5 mm, glabrous; style branches 8–10. Fruit semi-globose to broadly turbinate, 6–7 mm in diameter; mericarps 7–10, 2.5–3 mm excluding the arista, slightly striate near the base, eventually dehiscent, lateral walls usually thin, not veined, stellate puberulent, apex usually with (one or) two aristae, up to 1.5 mm long. Seeds reniform, about 2 mm long, blackish. Flowering during autumn–winter. This plant is widely distributed in tropical, subtropical, and warm temperate regions of the world, including North, Central, and South America and most of Africa and Asia. It grows as a weed, commonly found in disturbed areas, including urban zones. It flowers throughout the year. Chromosome number: 2*n* = 14 [37,38,39,40,41].

#### 3.1.13. *Sida rhombifolia* L. var. *rhombifolia*

Commonly known as arrowleaf sida, it is a variable, erect, perennial shrub or subshrub, reaching about 1 m in height. It is characterized by its rhomboid-shaped, alternate leaves that are serrated on the upper half and smooth on the lower half. The plant has small, yellow or white, axillary flowers that are solitary or in pairs, and a ribbed capsule fruit. It is common in the plains of Peninsular India. It occurs along roadsides and wastelands, generally at sea level. It flowers almost throughout the year. Chromosome number: 2*n* = 14 [42,43,44].

#### 3.1.14. *Sida rhombifolia* subsp. *retusa* (L.) Borss. Waalk.

A perennial, woody plant with upward branches, usually reaching 1.5 to 2 m in height. The leaves of this plant are rhombic to narrowly elliptic or obovate, with toothed margins at the top and attenuate at the base. The flowers are solitary or in pairs, axillary, and typically yellow. The fruits are capsules with mericarps that may be glabrous or spiny. It is a pantropical species, native to temperate and tropical regions of Asia. However, it has been introduced in West Africa, and Central and South America. It can be found in tropical and subtropical regions, in open areas such as roadsides, lawns, wastelands, coconut plantations, and scattered grassy areas, from sea level to altitudes of 1200 m. It flowers from October to December. reported [44].

#### 3.1.15. *Sida santaremensis* Monteiro

Subshrubs, up to 1 m tall. Erect stems, sparsely stellate-pilose, with hairs up to 0.5 mm in length. Leaves have petiole-free stipules, mononervate, linear, ca. 7 mm, subequal to the petiole; petiole ca. 3–10 mm, 1/5 of lamina length, densely stellate-pilose distally; leaf blades broadly elliptic to subrhombic, up to 5.5 cm long, 2–3.5 times longer than wide, smaller and narrower upward, base truncate to rounded, margins dentate almost to base, acute apex, surfaces uniformly stellate-pilose, densely stellate abaxially. Inflorescences are usually axillary, with solitary flowers; pedicels slender, up to 2 cm, generally three times longer than the calyx, two times longer than the underlying petiole. Flowers with acostillate calyx, ca 6–7 mm, stellate-pilose, triangular lobes; petals cream or pale yellow with a reddish spot at the base, 10 mm; staminal column glabrous; style 11-branched. Schizocarp fruits oblate-conical, 5–6 mm diam., subglabrous apically; mericarps 11, about 4–5 mm, dorsal wall somewhat sunken, lateral walls smooth to darkly reticulate, apex short-apiculate, with few hairs. *S. santaremensis* occurs in the subtropical and tropical Americas, from the southern United States (Florida) and Mexico to Colombia, Bolivia, Brazil, Paraguay, and Argentina, where it grows on sandy-stony soils, in disturbed environments, such as roadsides and wastelands [45,46].

#### 3.1.16. *Sida spinosa* L.

Shrubs or subshrubs to 1.5 m tall, erect with ascending branches, puberulous to pubescent by minute stellate trichomes. Leaf blades ovate-lanceolate, ovate-elliptic, or narrowly ovate, measuring 1–5.5 × 0.2–2 cm, acute or obtuse, with rounded base, truncate or somewhat cordate, and serrulate margin, discolored, with the upper side puberulate to pubescent with 2–6(–8)-radiate trichomes, the underside pubescent by stellate trichomes; petiole 0.3–2 cm long, commonly with a minute acute or spiniform projection at the base; subulate stipules, about 3–6 mm long. Axillary inflorescences uniflorous, commonly clustered near the apex of branches; pedicel 0.3–1.4 cm long (−2 cm in fruit), slender, articulated in the distal half, puberulous to pubescent; calyx 4–5.5 mm long, ca. ½ lobed, pubescent; lobes triangular, acute or apiculate; petals 5–6 × 3.5–4 mm, yellow or pale orange-yellow, the nerves sometimes reddish; staminal column measuring 1.5–2 mm long; filaments and anthers yellow; style with five yellow branches ca. 1.5 mm long; yellow stigmas. Schizocarp fruits conical to subglobose, 4–4.5 × 4–5 mm (spines included); mericarps 5, with two subapical spines 1.5 mm long, reticulate dorsally and laterally, puberulent towards the apex and on spines by simple 2–3-radiate antrorse trichomes. Seed are reniform, about 2 mm long, brownish, smooth. Found from the central United States to central Argentina, commonly as weeds in deciduous forests, savannas, open areas, and disturbed vegetation; 0–1900 m; flowers throughout the year. Chromosome number: 2*n* = 14, 28 [20,37].

#### 3.1.17. *Sida tuberculata* R.E. Fr.

Erect herbs or shrubs, annual suffrutescent. Erect stems, branched and covered with soft hairs. Leaves alternate, ovate or elliptic, with a rough texture and toothed or lobed edges, serrate-dentate margin in the distal portion, entire in the proximal portion, stellate trichomes on both sides. Solitary flowers or in clusters of 2 to 6 per inflorescence, with creamy-pink petals; stamens numerous and protrude from the flower. Fruits schizocarp with eight glabrous mericarps, with two short edges in the distal part. Reniform seeds, glabrous. It is distributed in South America, mainly in Brazil, and occurs in illuminated places in dry, sandy, or semi-humid soils. Chromosome number: 2*n* = 28 [19,33,34].

### 3.2. Sida Genus Species Names, Uses and Descriptions

Table 1 includes relevant information about the *Sida* species described in the previous section, including, if available, the scientific name and its synonyms, common name(s), commercial and ethnomedical uses; for the latter, the parts of the plant and the preparation technique used are also provided.

### 3.3. Pharmacological Activities of Some Sida Species 

Even though the genus *Sida* comprises approximately 200 species, occurring worldwide and widely recognized in traditional medicine because of its multiple uses for treating various diseases in different cultures, only 17 of these species (8.5%) have been pharmacologically studied in preclinical trials, namely *S. cordifolia*, *S. acuta*, and *S. rhombifolia* [14,23,27]. Although some of their traditional uses have been validated, clinical trials in humans with these plants are scarce. The *Sida* species analyzed in this review showed different pharmacological activities, which are described in Table 2.

### 3.4. Phytochemistry

Phytoconstituents are chemical compounds synthesized by plants as defense mechanisms against biotic and abiotic stress; they are also responsible of their color, smell, and the therapeutic potential they possess. Most of these compounds have pharmacological properties and commercial applications as medicines, enzymes, preservatives, aromatizers, fragrances, cosmetics, and fuels. Phytochemical screening is an important tool to identify and isolate chemical compounds in different parts of plants (root, stem, leaves, etc.) that have medicinal and industrial value [14]. Only 9 of the 17 species of Sida initially selected have been chemically studied, leading to the isolation of several compounds. Table 3 lists some of them together with their reported pharmacological activities.

**Table 1:** General information on *Sida* genus species.

Species	Scientific Synonym	Common Name	Commercial Uses	Traditional Medicinal Uses and Application Methods	References
*Sida acuta* Burm.f.	*Sida carpinifolia* L.f. *Sida carpinifolia* Bourg. ex Griseb. *Sida planicaulis* Cav.	-Vavalisin de Filipinas -Malva del Brasil -Malva de caballo (en Cuba) -Ancoacha del Perú -Pickna del Perú.	N/R	Different parts of the plant are used for: Dandruff Rheumatism Hepatic disorders Kidney stones Nervous disorders Leaves prepared in “juice” are used to: Helminth management Vomiting Gastric problems The roots are employed as a treatment for: Respiratory diseases, such as tuberculosis and cough. Hemorrhoids Kidney inflammation Decrease fever Heart disease To cure the *empacho*	[32,47,48]
*Sida ciliaris* L.	*Sida fulva* A.St.-Hil. *Sida involucrata* A.Rich. *Sida muricata* Cav. *Sida longistipula* Merr. *Sida plumosa* Cav. *Sida tridentata* Cav. *Sida ononidifolia* Gand. *Sida microtricha* Gand. *Sida minutifolia* Gand. *Sida avicularioides* Gand. *Sida bellidifolia* Gand.	-Fan petals with fringes -Fan petals with bangs -Sida with fringes -Sida with bangs -Salmon Sida -Bishop’s cord -Huinar	N/R	This plant is used as a remedy for: Stomach and toothache Antiseptic agent Cure sores and general wounds Pharyngitis	[34,49]
*Sida cordata* (Burm f.) Borss. Waalk.	*Sida cordata* var. *cordata* *Sida cordata* var. *nasirii* Abedin *Lamarkia morifolia* Medik. *Melochia cordata* Burm.f. *Sida humilis* Cav. *Sida humilis* var. *veronicifolia* (Lam.) Mast. *Sida mathewsii* Turcz. *Sida morifolia* Cav. *Sida multicaulis* Cav. *Sida pilosa* Retz. *Sida radicans* Cav. *Sida retzii* J.F.Gmel. *Sida unilocularis* L’Hér. *Sida veronicifolia* Lam. *Sida veronicifolia* var. *humulis* (Cav.) K.Schum. *Sida veronicifolia* var. *multicaulis* (Cav.) Baker f.	In India: -Farid buti -Rajbala -Bhumibala -Shaktibala In Pakistan -Simak	N/R	Root and stem paste of this species is used: To remove pus from boils by external application. In Hindu medicine, is known as Siddha or Ayurveda and the roots are used as: Diuretic Astringent Remedy for stomach problems Febrifuge Demulcent Seeds are administered to serve as: Laxative Aphrodisiac Demulcent It is recommended against: Cystitis Colic Gonorrhea Hemorrhoids Other medicinal uses include: Neurological disorders such as hemiplegia, facial paralysis, sciatica, general weakness, and headache. Urinary problems Tuberculosis Diabetes Fever and rheumatism Uterine disorders	[14,50]
*Sida cordifolia* L.	*Malvinda cordifolia* (L.) Medik	-Bala -Country mallow -Flannel Sida -Flannel grass -Heart leaf Sida -Sida-White burr	As a forage plant, insulation raw material and cellulose source.	The plant is used as a curative for: Dysentery, diarrhea Bronchial asthma Cold Chills Fever Headache Cough, nasal congestion or wheezing Nasal congestion Edema Weight loss Malaria Urinary problems	[27,51]
*Sida cordifolia* L. subsp. *maculata* (Cav.) Marais	*Sida maculata* Cav.	Flannel weed	N/R	It is used in Indian, American, and Chinese medicinal systems to treat conditions such as asthma, gonorrhea, nasal congestion, stomatitis, and inflammatory disorders.	[17,52]
*Sida hyssopifolia* C.Presl	*Sida callifera* Griseb. *Sida collina* Schltdl. *Sida corymbosa* R.E.Fr. *Sida costata* Schltdl.	Hyssop-leaved sida. Hyssop-leaf sida. Hyssop sida.	Used as animal food.	It is used in traditional medicine for treating diarrhea, dysentery, fevers and inflammations.	[23,39]
*Sida glutinosa* Comm. ex Cav.	*S. mysorensis* Wt. & Arn. *S. glabra*	-Smooth fanpetals -Sticky fanpetals -Cuban mallow -“*Escobita dulce*”	Stems provide textile fiber.	Roots and aerial parts of this plant are useful to treat: Pulmonary tuberculosis Rheumatism	[38]
*Sida javensis* Cav.	*Sida* *pilosa* Retz.	-Java golden flower noon (Taiwan) -Java yellow ripening (China)	N/R	This plant is utilized for its medicinal properties, the roots are the key component in treating conditions such as snake bites, rheumatic pains, tuberculosis, and malaria. Also, it is employed as a remedy for boils, fevers, heart disease, and hemorrhoids. Certain regions also use it to manage respiratory illnesses like asthma, pneumonia, and bronchitis	[38,39,53]
*Sida linifolia* Juss. ex Cav.	*Malva hirsuta* Aubl. *Sida angustissima* Miq. *Sida campii* Vell. *Sida graminifolia* Rich. *Sida linearifolia* Thonn. *Sida longifolia* Brandegee. *Sida viminea* Fisch. ex Link.	-*Trebol sabanero* (savannah clover) -Tongue of bird -Lancet leaf -Flaxleaf Fanpetals	Remediation of contaminated soils by metals due to their capacity to store nickel.	The infusion made from flowers and leaves is used as: Laxative Stomach reliever	[32,34,54]
*Sida mysorensis* Wight & Arn.	*Sida glutinosa* Roxb. *Sida urticifolia* Wight & Arn. *Sida wightiana* D.Dietr.	-Fan petals from Mysore (India) -Chinese muntjac (species of barking deer, Muntiacus reevesi)	Their fibers serve to reinforce polymeric compounds.	In Ayurvedic medicine, seeds are used to: Improve appetite Maharastra tribes (Indian western peninsular region) use leaves powder to: Heal wounds.	[41]
*Sida planicaulis* Cav.	*Malvastrum carpinifolium* (Medik.) A.Gray *Malvinda carpinifolia* Medik. *Sida acuta* subsp. *carpinifolia* (Medik.) Borss.Waalk. Sida *acuta* var. *carpinifolia* (Medik.) K.Schum. *Sida carpinifolia* L.f.	In Brazil: *guanxuma*, *chá da Índia*, *guaxima*, *malva brava*, *relógio de vaqueiro*, *vassoura*, *vassourinha*, *douradinha* *do campo*, *vassoura tupixá*, and *tupitixa*	It is considered an invasive weed in some areas, as it can negatively affect livestock due to its toxicity.	In Brazil, it is used to treat body pain. In India, it is traditionally used as a tonic, for urinary and blood disorders, and for liver and nervous system issues.	[22]
*Sida rhombifolia* L.	*Diadesma rhombifolia* Raf. *Malva rhombifolia* (L.) E.H.L.Krause *Napaea rhombifolia* (L.) Moench *Sida adusta* Marais. *Sida andicola* Gand. *Sida arbuscula* Zipp. ex Span. *Sida canariensis* Cav. *Sida canescens* Cav. *Sida compressa* DC. *Sida forsteri* Montrouz. *Sida fryxellii* Sivar. & Pradeep *Sida grata* Gand. *Sida hondensis* Kunth *Sida incerta* A.St.-Hil. & Naudin *Sida insularis* Hatus. *Sida kohautiana* C.Presl. *Sida nudata* Gand. *Sida philippica* DC. *Sida praelonga* Gand. *Sida pringlei* Gand. *Sida recisa* Link *Sida retusifolia* Stokes	-Arrowleaf sida -Big Jack -Cuban jute -Indian Hemp -Paddy lucerne -Queensland hemp -Rhombus-leaved sida -Sida Hemp -*Escobilla* -*Malvilla* -*Malva amarilla* -*Tlalamate* -*Malvavisco* -*Naranjillo* -*Oreja de burro*	Sourcing of high-quality natural fibers to produce textiles and handicrafts.	In Mexico, it is traditionally used in different states to treat: Oaxaca Rectal baths “*Susto*” in English scare Epilepsy Nerves Fatigue and weakness in children Hungry Courage or muin Toothache Veracruz The “latido” (heartbeat) Gingivitis Inflamed belly ”Empacho”, diarrhea Cough Hair loss Weakness Morelos Gingivitis Puebla A dough made with leaves are used for: Aphthas (Postemillas) Infusions made from the branches are used for: Stomach pain, gastritis and/or stomach ulcers With the root “agua de tiempo” (water at room temperature) is prepared for: Dysentery A decoction made from branches or leaves for: Washes Macerated plant for: Emplasts Nayarit Macerated with beef fat is placed on a wipe for: Pimples or “born” (boils) Cure wounds as a disinfectant Prepared in other ways, it is used for: Animal bites Anti-crotalic Fever	[34,48,55]
*Sida rhombifolia* subsp. *retusa* (L.) Borss.Waalk.	*Sida rhombifolia* var. *retusa* (L.) Griseb. *Sida retusa* L. *Meximalva retusa* (L.) F.C.Ho	Janglimedhi (Hindi); Atibata, Kallam gadale (Kannada); Bala (Sanskrit); Athibala, Bala, Jangli methi (Marathi)	N/R	A decoction derived from the plant’s roots is highly employed by local Ayurvedic practitioners. They use it to treat rheumatism and a range of neurological conditions, such as epilepsy. Furthermore, it acts as a diuretic for urinary calculus issues and as an antipyretic for fevers that involve shivering and convulsions.	[56]
*Sida rhombifolia* L. var. *rhombifolia*	*Sida rhombifolia* L. var. *rhombifolia* *Sida rhomboidea* Roxb. *Sida rhombifolia var. rhomboidea* (Roxb.) Mast *Sida adjusta* Marais *Sida alba* Cav. *Sida canariensis* Cav.	Arrow leaf sida, Cuban jute, Indian hemp, Broom weed	N/R	Pounded leaves of the plant are applied as a paste to reduce swelling and as a cure for boils and headaches. Root decoction is taken as tea to treat diarrhea. In India, the plant is used in the treatment of gonorrhea. In Europe it is used as antitubercular agent. Decoction of the plant is used to treat rheumatic pain, cardiac problems and biliary problems in children. Fresh plant juice is used as demulcent and diuretic.	[57,58]
*Sida santaremensis* Monteiro	*Sida glaziovii* *Sida rhombifolia* var. *Subtomentosa* *Sida santaremensis* var. *krapovickasiana*	-Moth fanpetals -Brazilian Sida -*Guanxuma*	N/R	This species is useful to treat: Cough Fever Vascular diseases Hypertension	[45]
*Sida spinosa* L.	*Sida angustifolia* Lam. *Sida spinosa* var. *angustifolia* *Sida escobilla*	-*Escoba dura* (Hard broom) -*Huinar* -Malva de caballo (Horse mallow) -*Quesillo*	It is used to feed animals as well as for medicinal purposes.	In India it is used to treat: Ulcers Urinary and skin diseases Asthma Snake bites Arthritis Bronchitis Burning sensation Hemorrhoids Fever Weaknesses Decoction made from leaves is given for: Calming bladder and genitourinary tract irritation	[20,27]
*Sida tuberculata* R.E. Fr.	*Sida hyssopifolia* C. Presl	-*Escoba*	N/R	Commonly used to treat: Inflammation Diabetes Vascular disorders. Wound healing Against insect bites Analgesic Healing	[34,59,60]

N/R = Not reported.

**Table 2:** *Sida* genus species pharmacological activities.

Species	Pharmacological Activity	In Vitro Assay	In Vivo Assay	Extract and Plant Tissue	Dose or Concentration	Statistical Significance	Control	Reference
*S. acuta* Burm.f.	Antioxidant	Antioxidant enzymes glutathione reductase, superoxide dismutase, catalase and malonylaldehyde quantification to estimate lipid peroxidation in Wistar rats’ plasma.	Liver and kidney damage induced with carbon tetrachloride (3 mL/kg bw) and rifampicn (250 mg/kg bw).	Leaves ethanolic extract, administered o.p.	50 and 100 mg/kg	*p* < 0.05.	Silymarin (100 mg/kg bw.).	[61]
	Antimicrobial	*P. falciparum* drug sensitivity in vitro assay.		Oleanolic acid and cryptolepine isolated from the EtOAc-soluble fraction of whole-plant hydroalcoholic extract.	3 µg/mL	N/R	Artemisinin (250 μg/mL).	[62]
		NCCLS microdilution method.		Methanolic from roots.	1800 μg/mL	*p* < 0.05	N/R	[63]
		Standard inhibition zone method against Gram-positive and Gram-negative pathogens.		Aqueous leaves extract. Alkaloid fraction obtained from aqueous leaves extract.	20, 40 and 60 µg/mL 2000 µg/mL	N/R N/R	Amoxicilin. Penicillin 10 UI, sulfadiazin 0.25 mg and spectinomycin 100 µg.	[63] [64]
		Minimal inhibitory concentration test against Gram-negative bacilli strains.		Leaf ethanolic extract.	8 mg/mL	*p* < 0.05	Gentamicin.	[65]
	Anticarcinogenic	3-(4,5-dimethylthiazol-2-yl)-2,5-diphenyltetrazolium bromide (MTT) assay against human breast cancer cells, ATCC^®^ HTB-26™		Methanolic and aqueous aerial parts extracts.	25, 50, 75, 100 and 1000 µg/mL (MeOH); 100, 250, 500, 750 and 1000 µg/mL (Water).	*p* < 0.05	DMSO (0.1%).	[66]
	Anti-lipooxygenase	In vitro enzyme inhibition as well as molecular docking studies.		Crude ethanolic extract from whole plant.	40 mg/mL.	N/R	Nordihydroguaiaretic acid (100–600 mg/mL)	[67]
	Antiviral	MTT assay against MCF-7 tumor cells compared to a cisplatin reference drug, as well as against the normal LLC-Mk2 cell line.		Methanolic extract from whole plant.	100 mg/mL	N/R	Cells with no virus (SV, herpes simplex virus; SINV, Sindbis virus or poliovirus type 1), and cells infected with untreated virus.	[68]
	Antihyperglycemic	Mammalian and yeast α-glucosidase inhibition assay.		Acetonic from aerial parts.	100 µg/mL^−1^	*p* ≤ 0.05 and *p* ≤ 0.01.	Acarbose and quercetin.	[69]
	Anti-inflammatory		TPA-induced ear edema assay in mice.	Acetonic from aerial parts.	1 mg/ear	*p* ≤ 0.05 and *p* ≤ 0.01.	Indomethacin (0.10 mg/ear).	[69]
	Analgesic		Acetic-acid-induced writhing test and the formalin test in mice.	Aqueous and acetone extract.	200, 400 y 600 mg/kg.	*p* ≤ 0.05.	Paracetamol (100 mg/kg).	[70]
	Antinociceptive		Formalin-induced nociception (plantar surface) in mice.	Aqueous and acetone extract.	200, 400 y 600 mg/kg.	*p* ≤ 0.05.	Paracetamol (100 mg/kg).	[70]
	Anxiolytic		Elevated plus maze (EPM) and open-field tests (OFT) in CD1 mice.	Ethanolic extract from leaves and stems.	50, 100, 300, and 500 mg/kg.	*p* < 0.05.	Diazepam at 1 mg/kg (EPM) as well as 1 and 5 mg/kg (OFT).	[71]
	Antionvulsivant		PTZ-induced seizures (75 mg/kg) in CD1 mice.	Ethanolic extract from leaves and stems.	500 mg/kg.	*p* < 0.05.	Diazepam (1 mg/kg).	[71]
	Sedative		Sodium pentobarbital-induced sleeping time test in CD1 mice.	Ethanolic from leaves and stems.	500 and 1000 mg/kg.	*p* < 0.05.	Diazepam (1 mg/kg).	[71]
	Antipyretic		Yeast suspension-induced pyrexia in Wistar rats.	Petroleum ether, acetonic, ethanolic, and aqueous from leaves.	500 mg/kg.	*p* < 0.05.	Paracetamol (30 mg/kg).	[72]
*S. ciliaris* L.	Photoprotective	In vitro solar photoprotection assays to quantify UV radiation.		Hexane, ethanol, and methanol fractions from aerial parts.	500 and 1000 µg/mL.	N/R	Combination of the p-aminobenzoic acid ester (7% octyl dimethyl p-aminobenzoic acid) and the benzophenone derivative (3% oxybenzone).	[73]
*S. cordata* (Burm f.) Borss. Waalk.	Antioxidant	In vitro antioxidant assays in blood samples.	STZ-induced diabetes (55 mg/kg^−1^) in Wistar rats.	Ethanolic extract from aerial parts.	200 and 400 mg/kg.	*p* < 0.05.	Glibenclamide (5 mg/kg).	[74]
		In vitro antioxidant assays.		Methanolic extract from roots.	10% *w*/*v*	N/R	Ascorbic acid and/or Trolox (10–1000 µg/mL).	[75]
		In vitro antioxidant, anti-lipid peroxidation, and phosphomolybdate assays in blood, pancreas, liver, and testes homogenates.	Alloxan-induced diabetes in Sprague–Dawley rats	Methanolic extract from the entire plant and its hexane, ethyl acetate, n-butanol, and aqueous fractions.	150 and 300 mg/kg.	*p* < 0.05.	Glibenclamide (5 mg/kg).	[76]
	Nephroprotective	In vitro antioxidant enzymes activities quantification and lipid peroxidation (TBARS) in renal homogenates.	CCl4-induced toxicity model (1 mL/kg) in Sprague–Dawley rats.	Ethyl acetate fraction derived from the whole plant methanolic extract.	150 and 300 mg/kg.	*p* < 0.05.	Silymarin	[77]
	Antidiabetic		Alloxan-induced diabetes in Sprague–Dawley rats.	Ethyl acetate fraction isolated from the whole-plant methanolic extract.	150 and 300 mg/kg.	*p* < 0.05.	Glibenclamide (5 mg/kg).	[76]
	Anti-lipoxygenase	In vitro enzyme inhibition as well as molecular docking studies.		Crude ethanolic extract from whole plant.	40 mg/mL.	N/R	Nordihydroguaiaretic acid (100–600 mg/mL).	[67]
	Gastroprotective		Indomethacin-induced gastric ulcer in rats.	Ethanolic extract from leaves.	200 y 400 mg/kg.	*p* < 0.001.	N/R	[78]
	Anti-asthmatic	Isolated goat tracheal chain preparation.	Clonidine-induced catalepsy in mice.	Ethanolic extract from leaves.	100, 200 and 400 mg/kg.	*p* < 0.001.	Histamine at 100 µg/mL (in vitro) and chlorpheniramine maleate (in vivo).	[78]
	Antibacterial	*S. aureus*, *E. coli* and *P. aeruginosa* growth inhibition assays in cultures.		Ethanolic, aqueous and chloroformic from roots.	200 y 400 mg/kg.	*p* < 0.001/*p* < 0.01.	Ampicillin	[79]
	Hepatoprotective	In vitro assessment of liver markers. Lipid peroxidation, and estimation of serum enzymes and bilirubin.	CCl4-induced acute liver toxicity in rats	Hydroalcoholic extract from leaves.	100, 200, and 400 mg/kg.	*p* < 0.05 or *p* < 0.001.	Silymarin (100 mg/kg).	[80]
*S. cordifolia* L.	Antioxidant	DPPH and FRAP (ferric reducing antioxidant power) free radical scavenging in vitro assays.		Methanolic extract from roots.	10% *w*/*v.*	N/R	Ascorbic acid and/or Trolox (10–1000 µg/mL).	[75]
		In vitro DPPH assay.	In vivo methods in wild-type *S. cerevisiae* BY 4743 (WT) and knock-out strain (Δtrx2) against H2O2-induced stress mediated damages.	Ethyl acetate, methanol, and water extracts from aerial parts.	100 µL (in vitro) and (0.4, 0.8, 1.6 mg/m (in vivo).	*p* < 0.01 and *p* < 0.001.	Ascorbic acid (10 mM in vivo and 0.2–1.0 mg/mL in vitro).	[81]
	Antidiarrheal		Castor oil-induced diarrhea, magnesium sulphate-induced diarrhea in Wister albino rats.	Hydroalcoholic extracts from roots.	100, 200, and 400 mg/kg.	*p* < 0.001.	Loperamide (5 mg/kg).	[82]
	Wound healing		Dexamethasone-induced (1 mg/kg) retardation of wound healing in rats.	Different hydrogel formulation mixed with ethyl acetate, methanol, or aqueous extracts from aerial parts.	2.5%	*p* < 0.01 and *p* < 0.05.	SSDeeUltra (silver sulphadiazine/chlorhexidine gluconate/*Aloe vera*)	[81]
	Analgesic		Acetic acid-induced pain model and hot plate method in mice.	Ethyl acetate and methanol extracts from aerial parts.	150, 300, and 600 mg/kg.	*p* < 0.001.	Aspirin (100 mg/kg) and morphine (4 mg/kg).	[51]
			Acetic-acid-induced writhing test and the formalin test in mice.	Aqueous and acetone extract.	200, 400, and 600 mg/kg.	*p* ≤ 0.05.	Paracetamol (100 mg/kg).	[70]
	Anti-lipoxygenase	In vitro enzyme inhibition and molecular docking studies.		Crude ethanolic extract from whole plant.	40 mg/mL.	N/R	Nordihydroguaiaretic acid (100–600 mg/mL).	[67]
	Anti-inflammatory		Carrageenan-induced paw edema in rats.	Ethyl acetate and methanol extracts from aerial parts and roots.	150, 300, 600 mg/kg.	*p* < 0.001.	Indomethacin (6 mg/kg).	[51]
			Quinolinic-acid-induced neurotoxicity (55 µg/100 g bwt/day) in rats.	Ethanolic extract from roots.	50 mg/100 g bwt/day.	*p* < 0.05.	Deprenyl (100 µg/100 g bwt/day).	[83]
	Antibacterial	Minimum inhibitory/bactericidal concentrations (MIC/MBCs) for Gram-positive and negative bacterial strains assay.		Hexane, chloroform, methanol, and aqueous crude extracts from roots.	8000 to 0.003 μg/mL	N/R	Methicillin-resistant *S. aureus* and *S. epidermidis*.	[84]
	Hypoglycemic		Glucose tolerance test in 18 h fasted rats.	Methanol extract from aerial parts and roots.	600 mg/kg	*p* < 0.05.	Normal rats.	[51]
	Antidiabetic		STZ-induced diabetes (55 mg/kg^−1^) in Wistar rats.	Ethanol extract from aerial parts.	200 and 400 mg/kg.	*p* < 0.05.	Glibenclamide (5 mg/kg).	[74]
	Antiperoxidative	In vitro hydroperoxide estimation by Mair and Hall’s method in quinolinic-acid-induced neurotoxicity in rats.		Ethanolic extract from roots.	50 mg/100 g bwt/day.	*p* < 0.05.	Deprenyl (100 µg/100 g bwt/day).	[83]
	Anti-arthritic		Patients with knee osteoarthritis (joints) administered with Ayurvedic preparation for 30 days (o.p).	*Bala moola churna ksheerapaka* (medicated milk) from roots Ayurvedic medicine.	80 mL	*p* < 0.05.	N/R	[85]
	Antihyperlipidemic	Tissue damage biomarker estimation (total cholesterol, triglycerides, low density lipids, plasma creatinine, plasma urea nitrogen).		Ethanol extract from aerial parts.	200 y 400 mg/kg.	*p* < 0.05.	Glibenclamide (5 mg/kg).	[74]
	Immunomodulatory	LPS-induced cytokine expression estimation on splenocytes, macrophages and RAW 264.7.		Aqueous extract from roots.	100 ng/mL.	*p* < 0.001.	LPS-untreated cells.	[86]
	Cytotoxic	MTT assay on human breast cancer (MCF7), ovarian cancer (PA1), colon cancer (HT29), melanoma (A375), liver cancer (HepG2), and normal mouse embryonic fibroblast (NIH3T3) cell lines.		Ethanolic extract.	3.125, 6.25, 12.5, 25, 50 and 100 μg/mL	*p* < 0.05.	Cisplatin and 5-Fluorouracil for HT29 cells, both at (3.125, 6.25, 12.5, 25, 50, and 100 μg/mL).	[86]
*S. cordifolia* L. subsp. *maculata* (Cav.) Marais	Antioxidant	Free radical scavenging (DPPH) assay.		Ethanol extract from whole plant and its hexane, chloroform, ethyl acetate, butanol fractions isolated.	Different concentrations within the range of 24.0 to 143.0 μg/mL.	*p* < 0.05.	Ascorbic acid and BHT with EC50 of 14.08 and 20.26 µM, respectively.	[87]
*S. glutinosa* Comm. ex Cav.	Antioxidant	Free radical scavenging (DPPH) assay.		Glutinoside, 24(28)-dehydromakisterone A and chrysin isolated from the butanolic fraction obtained from the aerial parts.	1, 5, 10, 25, 50, 75 µg/mL.	N/R	BHT (*di*-*tert*-butylhydroxytoluene) at 50 and 100 µg/mL.	[88]
	Antifungal	Growth inhibition test on *F. oxysporum* strain medium microdilution in 96-well plates.		Pentyl-10,12 dimethyl-11-hydroxyoleate and kaempferol-5-*O*-β-D-(6”-*O*-trans coumaroyl)- glucopiranoside isolated from methanolic aerial parts extract.	100 μg/disc.	N/R	N/R	[89]
	Hepatoprotective	Estimation of glutamic-oxaloacetic transaminase, glutamic-pyruvic transaminase, alkaline phos-phatase, as well as glycerol kinase enzymes inhibition.		Glutinoside and 24(28)-dehydromakisterone A isolated from the aerial parts methanolic extract.	5, 10, 15, 20, and 25 µg/µL.	*p* ≤ 0.05.	Atorvastatin (10 µg/mL).	[88]
	Antibacterial	Kirby–Bauer technique against *E. coli* and *B. subtilis* strains.		Glutinoside and 24(28)-dehydromakisterone A isolated from the aerial parts methanolic extract.	10 µg/µL.	N/R	Gentamicin (10 µg/µL).	[90]
*S. hyssopifolia* C. Presl	Antihemorrhagic		Tail cut bleeding induction model in rats.	Methanolic of leaves.	250, 500, and 1000 mg/kg.	*p* ≤ 0.05/*p* < 0.01.	N/R	[91]
	Uterotonic	In vitro model of collagen-induced contraction in human uterine cells.		Aqueous from the whole plant	100–400 µg/mL.	*p* < 0.05.	Oxytocin (100 nM).	[92]
	Anti-ulcerogenic	Ethanol- and Diclofenac-induced gastric ulcer models in rats.		Aqueous leaf extract.	250, 500, and 1000 mg/kg.	*p* < 0.05.	Omeprazole (20 mg/kg).	[93]
	Wound healing		Wound excision model in Wistar rats.	Cream formulates with aqueous leaf extract.	2.5%, 5%, and 10%.	*p* < 0.05, *p* < 0.01, and *p* < 0.001.	N/R	[93]
	Anti-inflammatory		Albumin-induced paw edema in rats.	Aqueous leaf extract.	250, 500, 1000 mg/kg.	*p* < 0.05, *p* < 0.01, and *p* < 0.001.	Naprozen (5 mg/kg).	[93]
*S. linifolia* Juss. ex Cav	Anti-inflammatory	Protease, platelet aggregation, phospho-lipase A2 inhibition assays, albumin denaturation, membrane stabilization and heat-induced hemolysis in human red blood cells.		Ethanolic extract from leaf.	0.1, 0.2, 0.4, 0.6, and 0.8 mg/mL	*p* < 0.05.	Diclofenac, aspirin, and prednisolone.	[94]
	Antimalarial		Malaria-infected mouse model.	Ethanolic leaf extract.	100, 200, and 400 mg/kg.	*p* < 0.05.	Artesunate (80 mg/kg).	[94]
			Carrageenan and albumin-induced paw edema, formalin-induced arthritis tests in mice.	Ethanolic leaf extract.	200, 400, and 600 mg/kg.	*p* < 0.05.	Aspirin (100 mg/kg).	[95]
	Antinociceptive		Formalin-induced arthritis and acetic acid-induced writhing tests in mice.	Ethanolic leaf extract.	200, 400, and 600 mg/kg.	*p* < 0.05	Aspirin (100 mg/kg).	[95]
	Antioxidant	DPPH, ferric reducing power (FRAP), and total antioxidant capacity (TAC) assays; nitric oxide.		Ethanolic leaf fraction extract.	0.2, 0.4, 0.6, 0.8, and 1.0 mg/mL	*p* < 0.05.	Gallic acid, butylated hydroxytoluene, and ascorbic acid.	[95]
*Sida javensis* Cav.	Schistosomicidal		*Schistosoma mansoni*-infected mice model.	Aqueous from the whole plant.	40, 80, and 160 mg/kg.	*p* < 0.05.	Praziquantel (100 mg/kg).	[96]
				*n*-Butanol fraction from aerial aqueous extract.	8, 4, 2, 1, 0.5, and 0.25 mg/mL.	*p* < 0.05.	Penicilin (10,000 U/mL), streptomicyn (10,000 µg/mL), and amphotericin (25 µg/mL).	[97]
	Toxicological safety	Hematological and biochemical analysis on mice blood samples.	*Schistosoma mansoni*-infected mice model.	Aqueous from the whole plant.	Unique dose of 4, 8,12, 16, or 20 g/kg (oral acute); 400, 800, or 1600 mg/kg/8 d (sub-acute oral).	*p* < 0.05.	N/R	[96]
	Antioxidant	Radical scavenging DPPH assay.		Aqueous aerial extract and the ethyl acetate fraction obtained from it.	Five concentrations in the range of 25 to 200 μg/mL.	*p* < 0.05.	Rutin (2.5, 10, 15, 20, and 25 μg/mL).	[97]
	Hepatoprotective	Biochemical analysis to estimate the levels of malondialdehyde, lipid hydroperoxides, eosinophil peroxidase, myeloperoxidase, CAT, and SOD in mice liver homogenates.	*Schistosoma mansoni*-infected mice model.	Aqueous aerial extract (SpAE) and the *n*-butanol fraction (SpBF) obtained from it.	100, 200, and 400 mg/kg (SpAE), and 50, 100, and 200 mg/kg (SpBF).	*p* < 0.001.	Praziquantel (100 mg/kg).	[98]
	Antifibrotic	Determination of hepatic hydroxyproline and γ-interferon.	*Schistosoma mansoni*-infected mice model.	SpAE and SpBF.	100, 200, and 400 mg/kg (SpAE), and 50, 100, and 200 mg/kg (SpBF).	*p* < 0.05 and *p* < 0.001.	Praziquantel (100 mg/kg).	[98]
*S. mysorensis* Wight & Arn.	Antioxidant	DPPH and FRAP (ferric reducing antioxidant power) free radical scavenging in vitro assays.		Methanolic from roots.	10% *w*/*v*	N/R	Ascorbic acid and/or Trolox (10–1000 µg/mL).	[75]
	Lipoxygenase inhibition	In vitro enzymatic inhibition assays.		Whole-plant crude ethanolic extract.	40 mg/mL.	N/R	Nordihydroguaiaretic acid (100–600 mg/mL).	[67]
*S. planicaulis* Cav	Cytotoxic and genotoxic	MTT cell viability test in SH-5YSY cells.		Ethanolic leaf extracts.	4000, 2000, 1000, 500, 250, 125, 62.5, 31.25, 15.62, or 7 μg/mL.	*p* < 0.05.	DMSO (20%) and H_2_O_2_ (2 mM).	[99]
*S. rhombifolia* L.	Antioxidant	DPPH and FRAP in vitro assays.		Methanolic from roots.	10% *w*/*v*	N/R	Ascorbic acid and/or Trolox (10–1000 µg/mL).	[75]
		TLC-Bioautography method with DPPH reagent.		Methanol from leaves, stems, and roots.	10 mg/mL.	N/R	N/R	[100]
		DPPH, ABTS•+ and FRAP in vitro assays.		Volatile organic compounds isolated from leaves and stem of the plant.	8 mg/mL.	N/R	BHT and Trolox.	[101]
		DPPH in vitro assays.		Ethanolic from aerial parts.	200 mg/kg.	N/R	Ascorbic acid.	[102]
	Antihyperglycemic	Quantification of mammalian and yeast α-glucosidase enzyme activity.		Acetonic from aerial parts, trans-ferulate p-hydroxyphenethyl, and β-sitosteryl gluco-pyranosyl isolated from it.	100 µg/mL^−1^	*p* ≤ 0.05 and *p* ≤ 0.01.	Acarbose and quercetin.	[69]
	Anti-inflammatory		TPA-induced ear edema in mice.	*p*-hydroxyphenethyl *trans*-ferulate isolated from the hexanic aerial parts extract.	1 µmol/ear	*p* ≤ 0.05 and *p* ≤ 0.01.	Indomethacin (1 mg/ear).	[69]
			LPS-induced periapical inflammation in rats.	Ethanolic from roots.	0.6, 1.2, and 2.4 g/kg.	*p* < 0.05.	Diclofenac (1.2 g/kg).	[103]
		Nitric oxide inhibition in LPS-treated RAW 264.7 cell cultures.		Hexanic from the whole plant.	100 μg/mL.	*p* < 0.05.	A fresh culture medium.	[104]
			Carrageenin-induced paw edema in rat.	Ethanolic from aerial parts.	200 mg/kg.	*p* ≤ 0.05.	Indomethacin (10 mg/kg).	[102]
	Antinociceptive		Acetic acid-induced writhing and hot plate tests in mice.	Ethanolic and acetonic from aerial parts.	200 mg/kg.	*p* ≤ 0.05, *p* ≤ 0.01, or *p* ≤ 0.001.	Indomethacin (5 mg/kg) and morphine (10 mg/kg).	[102]
	Anti- cholinesterase	Acetylcholinesterase enzyme activity quantification assay.		Hexanic of the whole plant.	100 μg/mL.	*p* < 0.05.	Tacrine.	[104]
	Cytotoxic	Methyl thiazole tetrazolium (MTT) reduction assay against cancer cell lines.		Hexanic of the whole plant.	100 μg/mL.	*p* < 0.05.	N/R	[104]
	Vasorelaxant	Percent relaxation assay in rodent mesenteric arteries precontracted with phenylephrine.		Quindolinone and the salt of cryptolepine isolated from aerial parts ethanolic extract.	10^−12^–10^−3^ M.	*p* < 0.05.	N/R	[105]
	Antifungal	Determination of minimum inhibitory concentration.		10-methylcryptolepinone and 10-ethylcryptolepinone isolated from the ethanol crude extract.	64, 256 and 512 μg/mL^−1^.	N/R	Amphotericin B (32 μg/mL^−1^).	[106]
	Proapoptotic	Flow cytometry analysis and real-time PCR assay in HepG2 cells.		EtOAc extract of the leaf.	300 μg/mL.	*p* < 0.01 and *p* < 0.001.	Cells without extract treatment.	[107]
	Antiproliferative	MTT assay against HepG2 cells.		EtOAc extract of the leaf.	364.3 μg/mL.	*p* < 0.01 and *p* < 0.001.	Cells without extract treatment.	[107]
	Antidiarrheal		Castor oil-induced diarrhoea, Castor oil-induced enteropooling and gastrointestinal motility tests in rat.	Methanolic root extract.	200 and 400 mg/kg.	*p* < 0.05, *p* < 0.01, and *p* < 0.001.	Diphenoxylate (50 mg/kg) and Atropine sulphate (0.1 mg/kg).	[108]
	Antibacterial	Determination of minimum inhibitory concentration.		10-methylcryptolepinone and 10-ethylcryptolepinone isolated from the ethanol crude extract.	N/R	N/R	Gentamicin (64 μg/mL^−1^).	[106]
		Disk diffusion antimicrobial assay.		Methanolic root extract.	50 mg/mL.	N/R	Ciprofloxacin.	[109]
		Determination of zone of inhibition.		Aqueous-methanol aerial part extract.	250 and 500 mg/mL.	*p* < 0.05.	Chloroamphinicole (30 μg/mL).	[110]
	Anxiolytic		Elevatedplus maze model in mice.	Ethanolic extract of the whole plant.	300 mg/kg.	*p* < 0.01.	Diazepam (2 mg/kg).	[111]
	Nephroprotective	Biochemical analysis of diabetic rat blood samples.	NAD and STZ-induced diabetic nephropathy in rats.	Aerial parts hydroalcoholic extract.	200 mg/kg.	*p* < 0.05.	Pioglitazone (10 mg/kg).	[112]
*S. rhombifolia* subsp. *retusa* (L.) Borss.Waalk.	Antioxidant	DPPH and FRAP (ferric reducing antioxidant power) free radical scavenging in vitro assays.		Methanolic from roots.	10% *w*/*v*	N/R	Ascorbic acid and/or Trolox (10–1000 µg/mL).	[75]
	Hypnotic/sedative		Pentobarbital-induced hypnosis in mice.	Crude aqueous extract from roots.	3, 10, and 15 g/kg.	*p* < 0.01 and *p* < 0.001.	Chlorpromazine (10 mg/kg).	[76]
	Antipyretic		Yeast-induced pyrexia in rat.	Crude aqueous extract from roots.	5 and 10 g/kg.	*p* < 0.01 and *p* < 0.001.	Acetyl salicylic acid (150 mg/kg).	[76]
	Hypoglycemic		Streptozotocin (STZ)-induced diabetes in rats.	Aqueous extract of leaves.	200 and 300 mg/kg.	*p* < 0.05.	Glibenclamide (10 mg/kg).	[113]
	Hypolipidemic	Biochemical analysis of blood samples from mice receiving treatment for 14 days.		Aqueous extract of leaves.	200 mg/kg.	*p* < 0.05.	Fenofibrate (100 mg/kg).	[113]
	Anticancer		Diethylnitrosamine-induced preneoplasia in rats.	Methanolic seed extract	50 and 100 mg/kg.	*p* < 0.05 and *p* < 0.001.	N/R	[114]
*S. rhombifolia* L. var. *rhombifolia*	Antioxidant	Metal iron chelating, DPPH, TEAC, H_2_O_2_, O_2_, HO· and NO· radical scavenging activities in vitro assays.		Methanol from aerial parts.	50, 100, 200, 500, and 800 µg/mL.	*p* < 0.05.	Ascorbic acid	[115]
	Antinociceptive		Acetic acid-induced writhing test in rats.	Ethyl acetate from leaves.	200 mg/kg.	*p* < 0.01.	Acetylsalicylic acid (100 mg/kg).	[116]
	Anti-inflammatory		Carrageenin-induced edema in rat paw.	Butanolic leaves extract.	200 mg/kg.	*p* < 0.001.	Phenylbutazone (100 mg/kg),	[116]
*S. santaremensis* Monteiro	Immunomodulatory	LPS-stimulated mice macrophages.		kaempferol 3-O-β-D-glucosyl-6’’-α-L-rhamnoside isolated from ethanolic aerial parts extract.	3.125 and 100 μM	*p* < 0.01 and *p* < 0.001.	Non LPS-stimulated macrophages.	[117]
	Antileishmania	Evaluation of the leishmanicidal activity in vitro.		kaempferol 3-O-β-D-glucosyl-6’’-α-L-rhamnoside isolated from ethanolic aerial parts extract.	6.25–800 μg/mL^−1^	*p* < 0.01 and *p* < 0.001.	Amphotericin B (2 μg/mL).	[117]
	Vasorelaxant	Precontractions induced with L-phenylephrine hydrochloride (Phe) or KCl and in vitro evaluation of endothelial-derived factors associated with vasorelaxation on rat superior mesenteric artery rings.		Ethanolic aerial parts extract.	(10^−9^ to 10^−5^ mol/L).	*p* < 0.01 and *p* < 0.001.	L-NAME (100 µmol/L); indomethacin (10 µmol/L) and atropine (1 nmol/L).	[45]
*S. spinosa* L.	Antipyretic		Yeast-induced pyrexia in rats.	Aqueous from root.	400 mg/kg.	*p* < 0.01.	Aspirin.	[118]
	Antimicrobial	Antimicrobial activity through agar diffusion technique.		Aqueous from root.	50 and 75 μL.	N/R	N/R	[118]
		Antimicrobial activity through agar diffusion technique.		Ethanolic extract of whole plant.	100, 200, 300, 400, and 500 μg/disc.	N/R	Ciprofloxacin (5 μg/disc).	[119]
		Determination of minimum inhibitory concentration.		Ethanolic leaf extract	50, 100, 200, 300, 400, and 500 μg/disc.	N/R	Ciprofloxacin (5 µg/disc) and Amphotericin B (30 µg/disc).	[120]
	Anthelmintic		Anthelmintic screening using Indian adult earthworm (*Pheretima posthuma*).	Ethanolic extracts of leaves.	25, 50, 100 mg/mL.	*p* < 0.05.	Mebendazole (25, 50,100 mg/mL).	[121]
*S. tuberculata* R.E. Fr.	Anti-inflammatory		Carrageenan-induced peritonitis model.	Methanolic extract from leaves.	10–300 mg/kg^−1^	*p* < 0.05, *p* < 0.01, and *p* < 0.001.	Dexamethasone (0.5 mg/kg^−1^).	[122]
		In vitro assays to measure cytokine levels on rats’ knees with monosodium iodoacetate (MIA)-induced knee osteoarthritis.		Aqueous from leaves and roots plus photobiomodulation therapy (PBMT; 904 nm, 18 J/cm2).	5 mg/mL.	*p* < 0.05, *p* < 0.01, and *p* < 0.001.	Diclofenac (10 mg/kg).	[123]
	Antitumor	Cell viability assay against HepG2 and MCF-7 (tumor cell lines).		Methanolic extract from leaves and roots.	543.6–593.4 μg/mL^−1^.	*p* < 0.05.	Human leukocytes (non-malignant cell line).	[122]
	Anti-nociceptive		Acetic acid-induced abdominal writhes and formalin model in mice.	Methanolic extract from leaves.	100 mg kg^−1^.	*p* < 0.001.	Naloxone (1 mg kg^−1^).	[59]
	Antioxidant	DPPH, ABTS^+^, Nitrogen derivative species radical scavenging activities, TBARS, Deoxyribose and FRAP in vitro assays.		Hydroethanolic extracts from leaves.	0.015 mg/mL^−1^.	*p* < 0.05.	N/R	[59]
		In vitro biochemical analyses of samples from rats with osteoarthritis induced by monosodium iodoacetate (MIA).		Infusion from leaves.	30 mg/mL.	*p* < 0.05.	Diclofenac (10 mg/kg).	[124]

N/R = Not reported.

**Table 3:** Chemical compounds identified in the genus *Sida* L.

Isolated Compound	*Sida* Species	Extract and Plant Part	Reference	Pharmacological Activities	Reference
**Alkaloids**					
Cryptolepine	*S. acuta* Burm.f. and *S. rhombifolia* L.	Whole-plant hydroethanolic extract.	[62]	BACE1 and Aβ inhibition, anti- Alzheimer, antitumor, decreases topoisomerase I and II activities, vasorelaxant, antiproliferative, proapoptotic, antibacterial, among others.	[105,125]
	*S. rhombifolia* L.	Total alkaloidal fraction obtained from a crude ethanolic extract of aerial parts.	[106]		
		Raw material and derived herbal preparations.	[126]		
	*S. spinosa* L.	Petroleum-ether and ethanol extracts of aerial parts and roots.	[127,128]		
Vasicine	*S. cordata* (Burm f.) Borss. Waalk.	Ethyl acetate, ethanol, aqueous, and chloroform extracts from leaves and stems.	[129]	Anticholinesterase, anti-allergic, antibacterial, anti-inflammatory, uterotonic, cardioprotective, antiasthmatic, antioxidant, among others.	[130]
	*S. cordifolia* L.	Water-soluble alkaloid fraction from roots.	[131]		
	*S. rhombifolia* L.	Raw material and derived herbal preparations.	[126]		
	*S. spinosa* L.	Petroleum-ether and ethanol extracts of aerial parts and roots.	[127,128]		
	*S. tuberculata* R.E.Fr.	Methanolic extracts of leaves and roots.	[60]		
	*S. acuta*, *S. rhombifolia* subsp. *retusa*, *S. spinosa* L., *S. cordata* (Burm f.) Borss, *S. cordifolia* L.	Methanolic root extracts	[132]		
Vasicinone	*S. cordata* (Burm f.) Borss. Waalk.	Ethyl acetate, ethanol, aqueous, and chloroform extracts from leaves and stems.	[129]	Antioxidant, anti-inflammatory, neuroprotective, cytotoxic, anticancer, and proapoptotic.	[130]
	*S. cordifolia* L.	Methanolic root extracts.	[132]		
	*S. rhombifolia* L.	Raw material and derived herbal preparations.	[128]		
	*S. spinosa* L.	Petroleum-ether and ethanol extracts of aerial parts and roots.	[127,128]		
Vasicinol				Antibacterial, anti-inflammatory, antioxidant, anticholinesterase, and sucrase-inhibitory effects.	[130,133]
	*S. rhombifolia* L.	Raw material and derived herbal preparations.	[126]		
	*S. cordifolia* L.	Benzene extract of the air-dried powdered root.	[17]		
10 methylcryptolepinone 10-ethylcryptolepinone	*Sida rhombifolia* L.	Total alkaloidal fraction obtained from a crude ethanolic extract of aerial parts.	[106]	Antifungal.	[106]
Ephedrine	*S. cordata* (Burm f.) Borss. Waalk.	Ethyl acetate, ethanol, aqueous, and chloroform extracts from leaves and stems.	[129]	Stimulates alpha- and beta-adrenergic receptors; bronchodilator; decongestant; increases arterial blood pressure, cardiac index, stroke volume, and systemic vascular resistance.	[134]
	*S. cordifolia* L.	Water-soluble alkaloid fraction from roots.	[131,135,136]		
		Methanolic root and aerial extracts.			
		Hydroalcoholic aerial parts extracts.			
	*S. rhombifolia* L.	Hydroalcoholic aerial parts extracts.	[136]		
	*S. spinosa* L.	Ethanolic extract of the whole plant.	[137]		
Ψ-ephedrine	*S. cordifolia* L.	Water-soluble alkaloid fraction from roots.	[131]	Stimulates alpha-adrenergic receptors; increases breathing rate and blood pressure; accelerates heart rate; causes bronchodilatation; raises blood glucose levels; stimulates the CNS; and produces a sense of increased energy and improved mood.	[138]
	*S. rhombifolia* L.	Hydroalcoholic aerial parts extracts.	[139]		
	*S. spinosa* L.	Ethanolic extract of the whole plant.	[137]		
		Petroleum-ether and ethanol extracts of aerial parts and roots.	[128]		
N-methyl ephedrine and N-methyl pseudoephedrine	*S. cordata* (Burm f.) Borss. Waalk.	Ethyl acetate, ethanol, aqueous, and chloroform extracts from leaves and stems.	[131]		
Hypaphorine	*S. cordifolia* L.	Benzene extract of the air-dried powdered root.	[17]	Increases non-rapid-eye-movement sleep time; anti-inflammatory.	[140]
		Crude extract from the leaves.	[141]		
	*S. spinosa* L.	Aqueous and ethanolic extracts of leaves.	[127,128]		
**Flavonoids**					
Quercetin 3-(2Gxylosylrutinoside)	*S. rhombifolia* L. *S. acuta* Burm.f.	Petroleum ether, chloroform, acetone, ethanolic, and aqueous extracts from leaves.	[142]	Anti-neuroinflammatory effects against LPS-induced damage in N9 cells in vitro.	[143]
Manghaslin	*S. rhombifolia* L. and *S. acuta* Burm.f.			Cytotoxic activity on T24 and MRC5 cells.	[144]
Myricetin 7-Rhamnoside	*S. rhombifolia* L. *S. acuta* Burm.f.			Anti-photoaging, anticancer, antihypertensive, immunomodulatory, anti-inflammatory, antiallergic, and analgesic.	[145]
Isorhamnetin 3-O-[b-D-glucopyranosyl-(1->2)-a L-rhamnopyranoside]	*S. rhombifolia* L. *S. acuta* Burm.f.			Inhibition of AChE, α-amylase, and α-glycosidase enzymes; antioxidant, anti-Alzheimer, antidiabetic, and cytotoxic effects.	[146]
Peltatoside	*S. rhombifolia* L. *S. acuta* Burm.f.			Anti-inflammatory and antinociceptive activities.	[147]
Quercimeritrin	*S. rhombifolia* L. *S. acuta* Burm.f.			Antioxidant, vasorelaxant, and α-glucosidase enzyme inhibition.	[144]
Rutin	*S. rhombifolia* L. *S. acuta* Burm.f.			Antioxidant, anti-inflammatory, antidiabetic, antiapoptotic, neuroprotective, nephroprotective, and hepatoprotective, among others	[148]
	*S. rhombifolia* L.	Hydroalcoholic crude extract from leaves.	[149]		
	*S. acuta* Burm.f.	Ethanolic leaf extract.	[150]		
Kaempferol	*S. acuta Burm.f.*	Ethanolic leaf extract.	[150]	Antioxidant, anti-inflammatory cardioprotective, neuroprotective, hepatoprotective, antidiabetic; promotes eye, skin, and respiratory system health.	[151]
	*S. rhombifolia* L.	Crude ethanolic extract from aerial parts.	[105]		
Kaempferol-3-(6-p-Coumaroyl) glucopyranoside.	*S. tuberculata* R.E.Fr.	Methanolic extracts of leaves and roots.	[60]	Antioxidant and proapoptotic activities.	[152]
Kaempferol-3-O-β-D-glucose-6″-α-D-rhamnose	*S. rhombifolia* L.	Crude ethanolic extract from aerial parts.	[105]		
Tiliroside	*S. rhombifolia* L.	Whol-plant hydroethanolic extract.	[62]	Antioxidant, anti-obesity, anti-diabetic, anti-inflammatory, and analgesic.	[152]
Quercetin	*S. cordifolia* L.	Petroleum ether and ethanol extracts from leaves.	[152]	Antioxidant, vasorelaxant, anti-inflammatory, neuroprotective, and inhibits the α-glucosidase enzyme.	[153]
	*S. acuta* Burm.f.	Ethanolic leaf extract	[150]		
	*S. linifolia* Juss. ex Cav	Ethanolic leaf fraction.	[150]		
	*S. rhombifolia* L.	Ethanolic leaves and stem extracts	[153]		
Isorhamnetin 3-O-[b-D-glucopyranosyl-(1->2)-a L-rhamnopyranoside]	*S. rhombifolia* L. and *S. acuta* Burm.f.	Petroleum ether, chloroform, acetone, ethanolic, and aqueous extracts from leaves.	[142]	Cardiovascular and cerebrovascular protection, antitumor, anti-inflammatory, antioxidant, organ protection, anti-obesity.	[154]
**Terpenes**					
Oleanolic acid	*S. acuta* Burm.f. and *S. rhombifolia* L.	Whole-plant hydroethanolic extract.	[62]	Antidyslipidemic, antidiabetic, antiviral, anti-HIV, antibacterial, antifungal, anticancer, anti-inflammatory, hepatoprotective, gastroprotective, antiatherosclerotic, and antiplasmodial.	[155]
Ursolic acid				Anticancer, anti-inflammatory, antimicrobial, antidiabetic, cardiovascular protection, an-tihyperlipidemic, antifungal, antihyperuricemic, anti-obesity, antibacterial, antiviral, antiestrogenic, and other properties.	[155]
β-amyrin glucoside	*S. acuta* Burm.f. and *S. rhombifolia* L.	Whole-plant hydroethanolic extract.	[62]	Cytotoxic and antiplasmodial.	[156]
	*S. rhombifolia* subsp. *retusa* (L.) Borss.Waalk	Methanolic extract of leaves.	[157]		
Phytol	*S. cordata* (Burm f.) Borss	Whole-plant ethanolic extract.	[158]	Antimicrobial, anticarcinogenic, anti-teratogenic, cytotoxic, antitumor, anticonvulsant, anxiolytic, antidepressant, antinociceptive, and anti-inflammatory.	[159]
	*S. rhombifolia* subsp. *retusa* (L.) Borss.Waalk	Methanolic extract of leaves.	[157]		
Squalene	*S. rhombifolia* subsp. *retusa* (L.) Borss.Waalk		[157]	Antitumor, antioxidant, and emollient.	[160]
**Pheophytins**					
13^2^-hydroxypheophytin α	*S. acuta* Burm.f.	Hexane, acetone, and methanol from aerial parts.	[69]	Antibacterial activity against *S. aureus* strains and induces NQO-1 enzyme activity in liver cell lines.	[161]
**Phytosterols**					
β-sitosterol glucoside	*S. acuta* Burm.f. and *S. rhombifolia* L.	Hexane, acetone, and methanol from aerial parts.	[69]	Inhibits the mammalian enzyme α-glucosidase.	[69]
	*S. linifolia* Juss. ex Cav	Alcoholic root extracts.	[54,95]		
	*S. rhombifolia* L.	Whole-plant hydroethanolic extract.	[62]		
Daucosterol	*S. rhombifolia* L. var. *Rhombifolia.*	*n*-hexane soluble fraction of methanolic stem extract.	[162]	Chemopreventive, neuroprotective, antioxidant, anti-inflammatory, antidiabetic, immunoregulatory, and anticancer.	[163]
Stigmasterol	*S. rhombifolia* subsp. *retusa* (L.) Borss.Waalk.	Methanolic extract of leaves.	[114]	Antibacterial, anticancer, anti-inflammatory, neuroprotective.	[164]
	*S. cordata* (Burm f.) Borss. Waalk.	Petroleum ether, ethanol, chloroform, and acetone leaf extracts.	[165]		
β-sitosterol	*S. acuta* Burm.f. and *S. rhombifolia* L.	Hexane, acetone, and methanol from aerial parts.	[69]	Anti-inflammatory, anticancer, hepatoprotective, antioxidant, cardioprotective, antidiabetic, and mitigates cognitive impairment.	[166]
	*S. cordifolia* L.	Seed oil.	[131]		
	*S. rhombifolia* L.	Aerial hexane extract.	[69]		
	*S. rhombifolia* L.	Ethanolic extract of aerial parts.	[105]		
	*S. acuta* Burm.f. and *S. rhombifolia* L.	Whole-plant hydroethanolic extract.	[62]		
	*S. rhombifolia* subsp. *retusa* (L.) Borss.Waalk	Alcoholic root extracts.	[114]		
γ-sitosterol	*S. rhombifolia* subsp. *retusa* (L.) Borss.Waalk	Methanolic extract of leaves.	[157]	Antidiabetic, antiapoptotic, antihyperglycemic, anti-inflammatory; inhibits glucogenesis, among others.	[167]
	*S. cordata* (Burm f.) Borss. Waalk.	Petroleum ether, ethanol, chloroform, and acetone leaf extracts.	[165]		
**Ecdysteroids**					
20-hydroxyecdysone, 20-Hydroxyecdysone-3-O-b-D-xylose, and 20-Hydroxyecdysone-3-O-b-D-glycopyranoside	*Sida tuberculata* R.E.Fr.	Methanolic extracts of leaves and roots.	[60,168]	Antioxidant, hypoglycemic, cardioprotective, hepatoprotective, neuroprotective, anticancer, anti-inflammatory, vasorelaxant, among others.	[60,168]
20-hydroxyecdisone 20,22-monoacetonide.	*S. acuta* Burm.f. and *S. rhombifolia* L.	Hexane, acetone, and methanol from aerial parts.	[69]		
20-Hydroxy-24-hydroxymethylecdysone					
25-acetoxy-20-hydroxyecdysone 3-O-β-D-glucopyranoside					
**Phthalates**					
Di(2-etilhexil)phtalate	*S. acuta* Burm.f., *S. cordifolia* L.	Whole plant methanolic extract.	[67]	Antimicrobial, cytotoxic, anti-inflammatory, and anti-lipoxigenase.	[67]
**Coumarins**					
(E)-suberenol	*S. acuta* Burm.f. and *S. rhombifolia* L.	Whole-plant hydroethanolic extract.	[62]	Antiplasmodial, anticoagulant, antifungal, anti-inflammatory, and antioxidant.	[62,169]
Thamnosmonin	*S. acuta* Burm.f. and *S. rhombifolia* L.	Whole-plant or aerial-parts hydroethanolic extract and EtOAc-soluble fractions.	[62]	Antiplasmodial and cercaricidal; antioxidant, anti-ulcer, antimalarial, antidiabetic, and anticancer.	[62,169]
Xanthyletin	*S. acuta* Burm.f. and *S. rhombifolia* L.	Whole-plant or aerial-parts hydroethanolic extract and EtOAc-soluble fractions.	[62]	Cytotoxic, anti-inflammatory, antitumor, anti α–glucosidase, phytotoxic, and antibacterial.	[62,170]
Scopoletin	*S. rhombifolia* L.	Ethanolic extract of aerial parts.	[104]	Anti-cancer, antidiabetic, anti-inflammatory, cardioprotective, anti-neuroinflammatory, anti-AChE, hepatoprotective, among others.	[171]
	*S. acuta* Burm.f.	Ethyl acetate-soluble extract of the whole plant.	[172]		
Scoparone	*S. rhombifolia* L.	Crude ethanolic extract from aerial parts.	[104]	Anti-inflammatory, antioxidant, anti-apoptotic, antimicrobial, anticancer, anti-depressive, antinociceptive, anti-cholinesterase, anti-hypertensive, and anxiolytic.	[170]
**Fatty acids**					
Palmitic acid	*S. rhombifolia* L.	Aqueous extract from leaves and stems.	[101]	Anti-inflammatory.	[173]
	*Sida cordifolia* L.	Hydroalcoholic extract from leaves and roots.	[25]		
Oleic acid	*S. cordata* (Burm f.) Borss. Waalk.	Whole-plant ethanolic extract.	[158]	Antioxidant, improves endothelial dysfunction, hypocholesterolemic, and anti-inflammatory.	[174]
		Ethyl acetate, ethanol, aqueous, and chloroform extracts from leaves and stems.	[126]		
	*S. rhombifolia* L.	Aqueous extract from leaves and stems.	[101]		
Stearic acid	*Sida cordifolia* L.	Hydroalcoholic extract from leaves and roots.	[25]	Immunomodulatory.	[174]
Malvalic acid	*S. rhombifolia* L.	Leaves and stems.	[101]	Anti-inflammatory, antimicrobial, and hypotensive.	[25]
Linoleic acid	*S. rhombifolia* L.	Leaves and stems.	[101]	Promotes oxidative stress, cytotoxicity, and lipid peroxidation.	[175]
Myristic acid					
Nonanoic acid	*S. cordata* (Burm f.) Borss. Waalk.	Whole-plant ethanolic extract.	[158]	Antimicrobial, energy modulator.	[175]
Octadecanoic acid, ethyl ester.				Antiproliferative and proapoptotic.	[175]
Octadecadienoic acid 9,12,15-Octadecadienoic acid, methyl ester, (Z,Z,Z)- 9,12-Octadecadienoic acid, methyl ester, (E,E)-				Inhibits glucose production, immunomodulator, and anti-inflammatory.	[175]
Caffeic acid	*S. rhombifolia* L.	Hydroalcoholic extract from leaves.	[149]	Anti-inflammatory, antioxidant, and neuroprotective.	[176]
**Xanthones**					
1,6-dihydroxyxanthone	*S. acuta* Burm.f. and *S. rhombifolia* L.	Whole-plant hydroethanolic extract.	[63]	Antioxidant and anticancer.	[177]
**Ceramides**					
Rhombifoliamide	*S. acuta* Burm.f. and *S. rhombifolia* L.	Whole-plant hydroethanolic extract.	[62]	Antiplasmodial, antioxidant, anti-inflammatory, and antidiabetic.	[62]
**Phenolic compounds**					
Rosmarinic acid	*S. cordifolia* L.	Methanolic root extract.	[84]	Antioxidant, anti-inflammatory, neuroprotective, antibacterial, and anti-neuroinflammatory.	[178]
Ferulic acid derivatives	*S. acuta* Burm.f.	Leaf ethanolic extract.	[65]	Anticarcinogenic, anti-neuroinflammatory, hepatoprotective, antioxidant, and antidiabetic.	[179]
		Leaf aqueous extract.	[180]		
		Methanolic root extract.	[181]		
	*S. linifolia* Juss. ex Cav	Ethanolic leaf fraction.	[182]		
Gallic acid	*S. acuta* Burm.f.	Leaf ethanolic extract.	[65]	Anticancer, antiinflammatory, antiobesity, antioxidant, anti-arthritic, anti-asthmatic.	[183]
Chlorogenic acid	*S. linifolia* Juss. ex Cav	Ethanolic leaf fraction.	[182]	Hypolipidemic, antioxidant, neuroprotective, antiviral, antibacterial, and antifungal.	[183]
4-methoxy cinnamic acid Vanillic acid Ellagic acid Sinapic acid	*S. linifolia* Juss. ex Cav	Ethanolic leaf fraction.	[182]		
p-hydroxybenzoic acid	*S. acuta* Burm.f.	Leaf aqueous extract.	[180]	Antimicrobial, antialgal, antimutagenic, antiestrogenic, hypoglycemic, anti-inflammatory, anti-platelet aggregating, nematicidal, antiviral, antioxidant, etc.	[184]
Resveratrol	*S. acuta* Burm.f.	Leaf ethanolic extract. Leaf aqueous extract.	[65] [180]	Anti-obesity, cardioprotective neuroprotective, antitumor, antidiabetic, antioxidants, anti-age, anticancer, anti-inflammatory, vasculoprotective, antiobesity, among others.	[185]
p-hydroxyphenethyl trans-ferulate	*S. rhombifolia* L. and *S. acuta* Burm.f.	Aerial hexane extract.	[69]	Antioxidant, yeast and mammalian α-glucosidase inhibition.	[69]

## 4. Discussion

Even though the genus *Sida* has around 200 species, only 17 have been studied pharmacologically, corresponding to 8.5%. These studies have focused mainly on acute in vitro and in vivo models, and have shown that they have antioxidant and anti-inflammatory activities, among others. It would be beneficial to conduct pharmacological research using subchronic or chronic models to evaluate possible effects on diseases associated with oxidative stress and inflammatory responses, including antinociceptive, cytotoxic, and neuroprotective effects. These plants produce compounds such as terpenes, flavonoids, coumarins, and phenolic acids, among others, which have remarkable biological properties and are key components that underlie their pharmacological activity. However, the chemical-pharmacological data available to date are not sufficient to support clinical studies on chronic diseases. Medium- and long-term studies, as well as toxicological studies and treatment standardization studies, are also needed.

According to the evidence compiled and analyzed in this review, scientific interest in this genus could increase even more. Out of the 17 plants with documented pharmacological effects, 11 have antioxidant properties (*S. acuta*, *S. cordata*, *S. cordifolia*, *S. cordifolia* subsp. *Maculata*, *S. glutinosa*, *S. hyssopifolia*, *S. linifolia*, *S. javensis*, *S. mysorensis*, *S. rhombifolia*, *S. rhombifolia* supsp. r*etusa*, and *S. rhombifolia* L. var. *rhombifolia*) and 6 exhibit anti-inflammatory activity (*S. acuta*, *S. cordifolia*, *S. hyssopifolia*, *S. linifolia*, *S. rhombifolia*, and *S. tuberculata*), pathophysiological features associated with chronic degenerative diseases such as diabetes and rheumatoid arthritis, as well as those affecting the central nervous system such as anxiety and epilepsy. In this sense, it should be noted that, in the present review, the two species with the most biological activities were *S. acuta* and *S. rhombifolia*, which, besides being anti-inflammatory and antioxidant, also have hypoglycemic, anxiolytic, sedative, analgesic, and antiepileptic properties, processes that have a significant impact on public health [56,69,71,109]. However, these data are not sufficient to advance either of these two species to a clinical study, which represents a great opportunity. *S. rhombifolia* is perhaps the most studied species of the genus, with 53 publications referenced in PubMed [186]. Because of scientific reports and its use in traditional medicine on different continents and geographical areas, including Mexico, this plant is of great interest.

Some species of this genus possess secondary metabolites with various pharmacological activities. Scoparone, a coumarin with anxiolytic, anti-inflammatory, antioxidant, and hypolipidemic properties, is one of the compounds reported from *S. rhombifolia* [187], and scopoletin, which is antidiabetic and anti-inflammatory [171]. Alkaloids such as vasicine and vasicinone, which act as vasorelaxants and immunomodulators, have also been isolated from *S. acuta*, *S. cordata*, and *S. cordifolia* [132,188]. Phytol is a diterpene from *S. rhombifolia* that has anticonvulsant activity [159].

According to the previous paragraph, these compounds together with their pharmacological effects, suggest that species of the genus *Sida*, in particular *S. rhombifolia* L., are excellent candidates for further scientific study, both preclinical and clinical, emphasizing the importance of conducting an agroeconomic study to produce high-quality plant material with standardized active ingredients without affecting the natural populations that inhabit different ecosystems around the world, thus benefiting patients, biological resources, and society.

## 5. Conclusions

This review provides information about *Sida* species, including those not considered in other reviews, which emphasizes the importance of these plants, as they are distributed across all continents and belong to the natural and cultural heritage of different countries, including Mexico. There is an indissoluble link between traditional Mexican medicine and its culture, since it is supported by healing beliefs and practices that have been transmitted from generation to generation. It is more than just a remedy for diseases; it reflects Mexicans’ intrinsic relationship with nature, their community, and their spirituality, strengthening the social structure in communities where access to modern medical care is limited. However, despite their great cultural importance, the lack of scientific evidence supporting the efficacy and safety of many medicinal plants is a problem that must be addressed urgently. Mexico’s megadiversity represents a pharmacological and chemical treasure for future preclinical and clinical research aimed at developing phytopharmaceuticals useful for treating diseases considered public health problems in our country. As shown in this review, the genus *Sida* has been scarcely studied despite having a long history of ethnopharmacological use in different cultures around the world. Therefore, it is important to highlight that these species represent a biotic resource of special interest for guiding pharmacological, pharmaceutical, chemical, and medical research toward the clinical field, including the design of a sustainable method for obtaining plant material to avoid overexploitation of this natural resource.

## Data Availability

No new data were created or analyzed in this study. Data sharing is not applicable to this article.

## References

[1] Lara E.A., Fernández E., Zepeda-del-Valle J.M., Lara D.J., Aguilar A., VanDamme P. (2019). Etnomedicina en los altos de Chiapas, México. Bol. Latinoam. Caribe Plant Med. Aromat..

[2] Das U., Islam S. (2019). A review study on different plants in Malvaceae family and their medicinal uses. Am. J. Biomed. Sci. Res..

[3] Hardy K. (2021). Paleomedicine and the Evolutionary Context of Medicinal Plant Use. Rev. Bras. Farm..

[4] Ortega-Cala L.L., Monroy-Ortiz C., Monroy-Martínez R., Colín-Bahena O., Flores-Franco G., Luna-Cavazos M., Monroy-Ortiz R. (2019). Plantas medicinales utilizadas para enfermedades del sistema digestivo en Tetela del Volcán, Estado de Morelos, México. Bol. Latinoam. Caribe Plant Med. Aromat..

[5] Reeves H.M. (2006). Sahagún’s “Florentine codex,” a little known Aztecan natural history of the Valley of Mexico. Arch. Nat. Hist..

[6] Reimers E.A.L., Fernández E.C., Reimers D.J.L., Chaloupkova P., Del Valle J.M.Z., Milella L., Russo D. (2019). An Ethnobotanical Survey of Medicinal Plants Used in Papantla, Veracruz, Mexico. Plants.

[7] Navarrete-Linares F. (2008). Los Pueblos Indígenas de México.

[8] WHO (2023). https://www.who.int/es/news-room/questions-and-answers/item/traditional-medicine.

[9] Osuna-Torres L., Tapia-Pérez M.E., Aguilar-Contreras A. (2005). Plantas Medicinales de la Medicina Tradicional Mexicana para Tratar Afecciones Gastrointestinales. Estudio Etnobotánico, Fitoquímico y Farmacológico.

[10] Orozco-Martínez J., Lira-Saade R., Jiménez-Estrada M., Ávila-Acevedo J.G., Serrano-Parrales R., Hernández-Delgado T. (2020). Medicinal plants of Oaxaca, Mexico: Ethnobotany and antibacterial activity. Bol. Latinoam. Caribe Plant Med. Aromat..

[11] Li Y., Kong D., Fu Y., Sussman M.R., Wu H. (2020). The effect of developmental and environmental factors on secondary metabolites in medicinal plants. Plant Physiol. Biochem..

[12] Karade D., Vijayasarathi D., Kadoo N., Vyas R., Ingle P.K., Karthikeyan M. (2020). Design of novel drug-like molecules using informatics rich secondary metabolites analysis of Indian medicinal and aromatic plants. Comb. Chem. High. Throughput Screen..

[13] Sen T., Samanta S.K. (2015). Medicinal plants, human health and biodiversity: A broad review. Adv. Biochem. Eng. Biotechnol..

[14] Aminah N.S., Laili E.R., Rafi M., Rochman A., Insanu M., Tun K.N.W. (2021). Secondary metabolite compounds from *Sida* genus and their bioactivity. Heliyon.

[15] Walker C.C., Eggli U., Nyffeler R. (2023). Malvaceae. Dicotyledons: Rosids (Illustrated Handbook of Succulent Plants).

[16] Paul T.K., Nayar M.P., Nayar M.P., Thothathri K., Sanjappa M. (1988). Malvaceae. Fascicles of Flora of India, Fascicle 19.

[17] Kumar S., Kumar S., Vishnoi V.K., Kumar P., Maheshwari D.K. (2023). *Sida cordifolia* L.: Ethnobotany, Phytochemistry, Phytonanotechnology, and Commercial Application. Curr. Pharm. Biotechnol..

[18] Linnaeus C.V. (1753). Species Plantarum.

[19] Krapovickas A. (2007). Las especies de *Sida* Secc. Malacroideae (Malvaceae) del Cono Sur de Sudamérica. Bonplandia.

[20] Fryxell P.A., Rzedowski J., de Rzedowski G.C. (1993). Familia Malvaceae Fascicle 16 In Flora del Bajío y de Regiones Adyacentes.

[21] Flores-Franco G., Rangel-Altamirano M.G., Wehncke-Rodríguez E.V., Bonilla-Barbosa J., Cruz-Durán R., Valencia-A S. (2024). Flora nativa y vegetación de la Reserva de la Biosfera Sierra de Huautla, Morelos, México. Acta Botánica Mex..

[22] Brandão J.L., Baracho G.S., Sales M.F., Viegas Filho M.P. (2017). Synopsis of *Sida* (Malvaceae, Malvoideae, Malveae) in the state of Pernambuco, Brazil. Phytotaxa.

[23] Rodrigues F.C., Morais de Oliveira A.F. (2020). The genus *Sida* L. (Malvaceae): An update of its ethnomedicinal use, pharmacology and phytochemistry. S. Afr. J. Bot..

[24] Rodrigo A.P. (1944). Las especies argentinas y uruguayas del género *Sida*. (Malvaceae). Rev. Mus. La Plata Nva Ser. Secc. Bot..

[25] Ahmed H., Juraimi A.S., Hamdani M.S., Rafii Y.M., Aslani F., Omar D. (2017). Comparative phytotoxic effects of aerial and root aqueous extracts of *Sida cordifolia* L. on germination and seedling vigour performance of lettuce, tomato and carrot. Bangladesh J. Bot..

[26] Fernandes de Oliveira A.M., Sousa-Pinheiro L., Souto-Pereira C.K., Neves-Matias W., Albuquerque-Gomes R., Souza-Chaves O., Vanderlei-de Souza M.D.F., Nóbrega de Almeida R., Simões-de Assis T. (2012). Total Phenolic content and antioxidant activity of some Malvaceae family species. Antioxidants.

[27] Dinda B., Das N., Dinda S., Dinda M., SilSarma I. (2015). The genus *Sida* L.—A traditional medicine: Its ethnopharmacological, phytochemical and pharmacological data for commercial exploitation in herbal drugs industry. J. Ethnopharmacol..

[28] Dorado O. (1997). Inventario Florístico de la Sierra de Huautla, Morelos.

[29] Dorado O., Maldonado B., Arias D., Sorani V., Ramírez R., Leyva E., Valenzuela D. (2005). Programa de Conservación y Manejo Reserva de la Biosfera Sierra de Huautla.

[30] Chaachouay N., Zidane L. (2024). Plant-derived natural products: A source for drug discovery and development. Drugs Drug Candidates.

[31] Marhold K., Kučera J., Akopian J.A., Alves L.I.F., Alves W.S., Baracho G.S., Barros e Silva A.E., Batista F.R.C., Calado L.L., Cordeiro J.M.P. (2023). IAPT chromosome data 39—Extended version. Taxon.

[32] Fryxell P.A. (1988). Malvaceae of Mexico. Syst. Bot. Monogr..

[33] Lavia G.I., Fernández A., Krapovickas A. (2007). Cromosomas de especies americanas de *Sida* (MALVACEAE). Bonplandia.

[34] Rondón J.B. (2009). La subfamilia Malvoideae (Malvaceae s.l.) en el occidente del estado Sucre, Venezuela. UDO Agric..

[35] Kumar S., Kumari S., Chand-Gupta R. (2012). Cytological investigations of some polypetalous plants from District Sirmaur of Himachal Pradesh in the Western Himalayas, India. Chromosom. Bot..

[36] Exell A.W. (1961). Malvaceae. Flora Zambesiaca.

[37] Venkatesh K.H., Dinesh B., Venu N., Munirajappa (2015). Chromosome numbers and karyotype studies of few members of Malvales. Am. J. Phytomed Clin. Ther..

[38] Areces-Berazaín F., Fryxell P.A., Greuter W., Rankin Rodríguez R. (2007). Malvaceae. Flora de la República de Cuba, 13th Fascículo.

[39] García-Mendoza A., Meave J. (2011). Diversidad Florística de Oaxaca: De Musgos a Angiospermas.

[40] Singh A.K., Sahu R.K. (2014). Seedling Morphology and its Systematic Implications Within the Genus *Sida* L. (Malvaceae). Indian J. For..

[41] Huang-Hua R.S. (2007). *Sida Linnaeus*, Sp. Fl. China.

[42] Rao K.S., Swamy R.K., Kumar D., R. A.S., Bhat K.G. (2019). Flora of Peninsular India. https://indiaflora-ces.iisc.ac.in/FloraPeninsular/herbsheet.php?id=0&cat=7.

[43] Sivarajan V.V., Pradeep A.K. (1994). Taxonomy of the *Sida rhombifolia* (Malvaceae) complex in India. SIDA Contrib. Bot..

[44] Sharif-Shamima N., Sadia-Sultana D., Lubna A., Rabeya B., Zaman M.A., Sheikh-Shamimul A. (2003). Differential Fluorescent Chromosome Banding of Four *Sida* spp. (Malvaceae). Cytologia.

[45] Rufino-Arcanjo D.D., Muniz-Oliveira N.N.P., Ferreira-Filho E.S., Albuquerque da Costa D., Chaves M.H., Romão-Borges A.C., Pereira de Oliveira A., Meneses-Oliveira R.C. (2011). Vasorelaxant response induced by *Sida santaremnensis* H. Monteiro ethanol extract on rat superior mesenteric artery. AJB.

[46] Fontes de Sousa V., Soares de Oliveira V. (2019). *Sida santaremensis* (MALVACEAE): A new record for Paraíbastate, in caatinga domain, northeastern Brazil. Pesqui. BOTÂNICA.

[47] Andrade-Cetto A., Heinrich M. (2005). Mexican plants with hypoglycaemic effect used in the treatment of diabetes. J. Ethnopharmacol..

[48] Monroy-Ortiz C., Castillo-España P. (2007). Plantas Medicinales Utilizadas en el Estado de Morelos.

[49] Alarcón-Aguilar F.J., Román-Ramos R., Soumyanath A. (2006). Antidiabetic plants in Mexico and Central America. Traditional Medicines for Modern Times: Antidiabetic Plants.

[50] Adhikari D.C., Das B.D. (2018). Some medicinal plants uses in ethnical group from Biratnagar, Eastern, Nepal. Am. Sci. Res. J. Eng. Tech. Sci..

[51] Kanth V.R., Diwan P.V. (1999). Analgesic, anti-inflammatory and hypoglycaemic activities of *Sida cordifolia*. Phytother. Res..

[52] Baracho G.S., Agra M.D.F. (2024). New synonyms and lectotypifications in *Sida* (Malvaceae-Malveae) reveal the first record and extension of the distribution area of *Sida maculata* to Brazil. Phytotaxa.

[53] Felayati T., Rustiami H., Susandarini R. (2024). *Sida penambangensis* (Malvaceae), a new *Sida* species from East Java, Indonesia. Reinwardtia.

[54] Nwankwo N.E., Ezeako E.C., Nworah N.E., Ogara L.O., Oka S.A., Aham E.C., Joshua P.E., Nwiloh B.I., Ezike T.C., Ashiakpa N.P. (2023). Bioactive compounds anti-infammatory anti-nociceptive and antioxidant potentials of ethanolic leaf fraction of *Sida linifolia* L (Malvaceae). Arab. J. Chem..

[55] García-Regalado G. (2014). Plantas Medicinales de Aguascalientes.

[56] Thangam J., Shanthakumari G. (1971). Central nervous system effects of *Sida retusa* root. Jpn. J. Pharmacol..

[57] Digital Flora of Eastern Ghats. https://indiaflora-ces.iisc.ac.in/EasternGhats/herbsheet.php?id=2320&cat=4.

[58] Plants of the World Online (POWO). Royal Botanic Gardens, Kew: Kew, UK, 2024. https://www.plantsoftheworldonline.org/.

[59] Bai M.D.A., Rani S.P.S., Balachandran S., Jayakumar G. (2012). The use of *Sida* plant in the preparation of Nayapayam Kashayam. IJRAP.

[60] da Rosa H.S., Coelho I.S., da Silva M.D., Fernandes M.S., Bertelli P.R., Minetto L., Moura S., de Paula F., Santos A.R., Mendez A.S.L. (2018). *Sida tuberculata* extract reduces the nociceptive response by chemical noxious stimuli in mice: Implications for mechanism of action, relation to chemical composition and molecular docking. Phytother. Res..

[61] Ogunmoyole T., Falusi O.O., Oderinde F. (2022). *Sida acuta* leaf extract attenuates oxidants-induced animal model of nephrotoxicity and hepatotoxicity. Clin. Phytosci..

[62] Kamdoum B.C., Simo I., Wouamba S.C.N., Tchatat-Tali B.M., Ngameni B., Fotso G.W., Ambassa P., Fabrice F.B., Lenta B.N., Sewald N. (2022). Chemical constituents of two Cameroonian medicinal plants: *Sida rhombifolia* L. and *Sida acuta* Burm. f. (Malvaceae) and their antiplasmodial activity. Nat. Prod. Res..

[63] Karou S.D., Tchacondo T., Tchibozo M.A., Anani K., Ouattara L., Simpore J., de Souza C. (2012). Screening Togolese medicinal plants for few pharmacological properties. Pharmacogn. Res..

[64] Senthilkumar R.P., Bhuvaneshwari V., Malayaman V., Ranjithkumar R., Sathiyavimal S. (2018). Phytochemical screening of aqueous leaf extract of *Sida acuta* Burm.f. and its antibacterial activity. J. Eme Tech. Innov. Res..

[65] Smanthong N., Tavichakorntrakool R., Tippayawat P., Lulitanond A., Pinlaor P., Daduang J., Sae-Ung N., Chaveerach A., Phetcharaburanin J., Boonsiri P. (2022). Anti-proteus activity, anti-struvite crystal, and phytochemical analysis of *Sida acuta* Burm. F. ethanolic leaf extract. Molecules.

[66] Uysal S., Gevrenova R., Sinan K.I., Bayarslan A.U., Altunoglu Y.C., Zheleva-Dimitrova D., Ak G., Cengiz-Baloglu M., Etienne O.K., Lobine D. (2021). New perspectives into the chemical characterization of *Sida acuta* Burm. f. extracts with respect to its anti-cancer, antioxidant and enzyme inhibitory effects. Process Biochem..

[67] Preethidan D.S., Arun G., Surendran M.P., Prasanth S., Sabu A., Sadasivan C., Haridas M. (2013). Lipoxygenase inhibitory activity of some *Sida* species due to di(2-ethylhexyl) phthalate. Curr. Sci..

[68] Anani K., Hudson J.B., de Souza C., Akpagana K., Tower G.H., Arnason J.T., Gbeassor M. (2000). Investigation of medicinal plants of togo for antiviral and antimicrobial activities. Pharm. Biol..

[69] Arciniegas A., Pérez-Castorena A.L., Nieto-Camacho A., Kita Y., Vivar A.R.D. (2017). Anti-hyperglycemic, antioxidant, and anti-inflammatory activities of extracts and metabolites from *Sida acuta* and *Sida rhombifolia*. Quim. Nova.

[70] Konaté K., Bassolé I.H., Hilou A., Aworet-Samseny R.R., Souza A., Barro N., Dicko M.H., Datté J.Y., M’Batchi B. (2012). Toxicity assessment and analgesic activity investigation of aqueous acetone extracts of *Sida acuta* Burn f. and *Sida cordifolia* L. (Malvaceae), medicinal plants of Burkina Faso. BMC Complement. Altern. Med..

[71] Benjumea D., Gómez-Betancur I., Vásquez J. (2016). Neuropharmacological effects of the ethanolic extract of *Sida acuta*. Rev. Bras. Farm..

[72] Sharma R., Kumar S., Shrama D. (2012). Antipyretic efficacy of various extracts of *Sida acuta* leaves. Res. J. Pharm. Biol. Chem. Sci..

[73] Machado-De Queiroz W.A. (2022). Estudo Fitoquímico e Avaliação Biológica Pioneiros da Espécie *Sida ciliaris* L. (Malvaceae Sensu lato). Master’s Thesis.

[74] Ahmad M., Prawez S., Sultana-Raina R., Pankaj N.K., Kumar-Verma P., Rahman S. (2014). Anti-hyperglycemic, anti-hyperlipidemic and antioxidant potential of alcoholic-extract of *Sida cordifolia* (Areal Part) in streptozotocin-induced-diabetes in Wistar-Rats. Proc. Natl. Acad. Sci. India Sect. B Biol. Sci..

[75] Subramanya M.D., Pai S.R., Upadhya V., Ankad G.M., Bhagwat S.S., Hegde H.V. (2015). Total polyphenolic contents and in vitro antioxidant properties of eight *Sida* species from Western Ghats, India. J. Ayurveda Integr. Med..

[76] Shah N.A., Khan M.R. (2014). Antidiabetic effect of *Sida cordata* in alloxan induced diabetic rats. Biomed. Res. Int..

[77] Shah N.A., Khan M.R., Nigussie D. (2017). Phytochemical investigation and nephroprotective potential of *Sida cordata* in rat. BMC Complement. Altern. Med..

[78] Kamble S.D., Purane L.M., Devade O., Redasani V. (2024). In Vitro and In Vivo evalution of anti-asthmatic activity of leaves of *Sida veronicafolia* (Lam). RJPPD.

[79] Gulnaz A.R., Thabassum S., Salahuddin M., Savitha G. (2018). Biological activity and phytochemical screening of different extracts of *Sida cordata* (Burm.F.) borssum root. Indian. J. Clin. Anat. Physiol..

[80] Mistry S., Dutt K.R., Jena J. (2013). Protective effect of *Sida cordata* leaf extract against CCl(4) induced acute liver toxicity in rats. Asian Pac. J. Trop. Med..

[81] Kumar S., Lakshmi P.K., Sahi C., Pawar R.S. (2019). *Sida cordifolia* accelerates wound healing process delayed by dexamethasone in rats: Effect on ROS and probable mechanism of action. J. Ethnopharmacol..

[82] Shahed-Al-Mahmud M., Jahan T., Towhidul-Islam M. (2018). Antidiarrheal activities of hydroalcoholic extract of *Sida cordifolia* roots in Wister albino rats. Orient. Pharm. Exp. Med..

[83] Swathy S.S., Panicker S., Nithya R.S., Anuja M.M., Rejitha S., Indira M. (2010). Antiperoxidative and antiinflammatory effect of *Sida cordifolia* Linn. on quinolinic acid induced neurotoxicity. Neurochem. Res..

[84] Iqbal H., Wright C.L., Jones S., da Silva G.R., McKillen J., Gilmore B.F., Kavanagh O., Green B.D. (2022). Extracts of *Sida cordifolia* contain polysaccharides possessing immunomodulatory activity and rosmarinic acid compounds with antibacterial activity. BMC Complement. Med. Ther..

[85] Mathew M., Jayshree C., Jibi V., Nilima G. (2021). A phytochemical study of bala dwayam (*Sida cordifolia* & *Abutilon indicum* Linn.) And clinical evaluation of its moola churna ksheerapaka in Sandhigata vata with special reference to janu sandhi. IAMJ.

[86] Pratima M.B., Sunganthi V., Milind V.B., Kothai R. (2020). In vitro antiproliferative activity of ethanolic extract of *Sida cordifolia* Linn against various cancer cell lines. Al Ameen J. Med. Sci..

[87] Silva D.A., da Silva T.M.S., da Silva-Lins A.C., da Costa D.A., Cavalcante-Sobral J.M., Matias W.N., Vanderlei de Souza M.F., Braz-Filho R. (2006). Chemical constituents and antioxidant activity of *Sida galheirensis* Ulbr. (Malvaceae). Quim. Nova.

[88] Das N., Nath J., Dinda B. (2012). Antioxidant Phytochemicals from *Sida glutinosa*. J. Pharm. Res..

[89] Das N., Saha T., Dinda B. (2016). A new antifungal aliphatic fatty acid ester from the aerial parts of *Sida glutinosa*. Chem. Nat. Compd..

[90] Das N., Das M.C., Dinda B. (2014). Enzyme inhibitor and antimicrobial phytochemicals from aerial parts of *Sida glutinosa*. IJPPR.

[91] John-Africa L.B., Aboh M. (2015). Evaluation of the haemostatic activities of *Sida corymbosa* in rats. Br. J. Pharm. Res..

[92] Attah A.F., O’Brien M., Koehbach J., Sonibare M.A., Moody J.O., Smith T.J., Gruber C.W. (2012). Uterine contractility of plants used to facilitate childbirth in Nigeria ethnomedicine. J. Ethnopharmacol..

[93] John-Africa L.B., Yahaya T.A., Isimi C.Y. (2014). Antiulcer and wound healing activities of *Sida corymbosa* in rats. Afr. J. Tradit. Complement. Altern. Med..

[94] Nwankwo N.E., Ashiakpa P.N. (2024). Antimalarial potential of ethanol extract, and anti-inflammatory properties of flavonoid-, terpenoid-, and alkaloid-rich fractions of *Sida linifolia* L. Food Mater. Res..

[95] Nwankwo N.E., Okeke E.S., Nworah F.N., Ezeako E.C. (2023). Phytochemical composition and potential anti-inflammatory and antioxidant mechanisms of leaf extracts of *Sida linifolia* L. (Malvaceae). J. Herb. Med..

[96] Jatsa H.B., Endougou A.M.E., Kemeta D.R.A., Kenfack C.M., Tchuem-Tchuente L.A., Kamtchouing P. (2009). In Vivo antischistosomal and toxicological evaluation of *Sida pilosa* Retz on mice BALB/c. Pharmacologyonline.

[97] Jatsa H.B., Pereira C.A.J., Pereira A.B.D., Negrão-Corrêa D.A., Braga F.C., Maciel G.M., Castilho R.O., Kamtchouing P., Teixeira M.M. (2015). In vitro evaluation of *Sida pilosa* Retz (malvaceae) aqueous extract and derived fractions on *Schistosoma mansoni*. Pharmacol. Pharm..

[98] Jatsa H.B., Russo R.C., Pereira C.A., Aguilar E.C., Garcia C.C., Araújo E.S., Oliveira J.L., Rodrigues V.F., de Oliveira V.G., Alvarez-Leite J.I. (2016). Improvement of the liver pathology by the aqueous extract and the n-butanol fraction of *Sida pilosa* Retz in *Schistosoma mansoni*-infected mice. J. Ethnopharmacol..

[99] Selbach M.T., Scotti A.S., Feistel C.C., Nicolau C.C., Dalberto D., Dos Santos N.G., Borsoi G., Ferraz A.B.F., Grivicich I., de Souza G.M.S. (2021). Evaluation of the cytotoxic and genotoxic effects of *Sida planicaulis* Cav extract using human neuroblastoma cell line SH-SY5Y. J. Toxicol. Env. Health A.

[100] Ratna-Sari E., Suhatri N., Ismed F., Prima-Putra D. (2021). Inventory, morphological and antioxidant profile of the Sumatera Sidaguri (*Sida* Spp.) plants. Adv. Health Sci. Res..

[101] Xu Z., Gao P., Liu D., Song W., Zhu L., Liu X. (2022). Chemical composition and In Vitro antioxidant activity of *Sida rhombifolia* L. volatile organic compounds. Molecules.

[102] Kumar A., Mishra P.G., Tomar S.D., Pathak M., Pandey K.R., Singh L. (2023). Phyto-therapeutic potential of aerial part of *Sida rhombifolia* for antiinflammatory, antinociceptive, and antioxidant activity. Int. J. Pharm. Qual. Assur..

[103] Tanumihardja M., Natsir N., Mattulata I.K., Lukman M. (2016). Potent anti-inflammatory effect of root of sidaguri (*Sida rhombifolia* L) on rat periapical lesion model. IJTPR.

[104] Mah S.H., Teh S.S., Ee G.C.L. (2017). Anti-inflammatory, anti-cholinergic and cytotoxic effects of *Sida rhombifolia*. Pharm. Biol..

[105] Chaves O.S., Teles Y.C., Monteiro M.M., Mendes-Junior L.D., Agra M.F., Braga V.A., Silva T.M., Souza M.F. (2017). Alkaloids and phenolic compounds from *Sida rhombifolia* L. (Malvaceae) and vasorelaxant activity of two indoquinoline alkaloids. Molecules.

[106] Oliveira M.S., Chaves O.S., Cordeiro L.V., Gomes A.N.P., Fernandes D.A., Teles Y.C.F., da Silva T.M.S., Freire K.R.L., Lima E.O., Agra M.F. (2023). Indoquinoline alkaloids from *Sida rhombifolia* (L.) (Malvaceae) and antimicrobial evaluation of cryptolepinone derivatives. J. Braz. Chem. Soc..

[107] Ahmadi M., Ebrahimzadeh M.A., Rafiei A., Kardan M., Ebrahimi M.A. (2022). Sida rhombifolia exerts anti-proliferative and pro-apoptotic effects in human liver cancer HepG2 cells In Vitro. Asian Pac. J. Cancer Prev..

[108] Ranjan S.R., Shankar M.U., Kumar P.S., Saiprasanna B. (2011). Evaluation of antidiarrhoeal activity of *Sida rhombifolia* Linn. root. Int. Res. J. Pharm..

[109] Saini A.K., Velumani A.N., Sakarkar S.N., Singh B.K., Rizwan M., Gupta A.A. (2023). Antibacterial activity of compounds extracted from *Sida rhombifolia* Linn. Bull. Environ. Pharmacol. Life Sci..

[110] Debalke D., Birhan M., Kinubeh A., Yayeh M. (2018). Assessments of Antibacterial effects of aqueous-ethanolic extracts of *Sida rhombifolia*’s aerial part. Sci. World J..

[111] Sundaraganapathy R., Niraimathi V., Thangadurai A., Jambulingam M., Narasimhan B., Aakash D. (2013). Phytochemical studies and pharmacological screening of *Sida rhombifolia* Linn. Hygeia J. D Med..

[112] Imran M., Robert S.M.J., Sharma M., Aeri V. (2023). Evaluation of *Sida cordifolia* and *Sida rhombifolia* extracts in a rat model of streptozotocin-induced diabetic nephropathy. Polim. Med..

[113] Dhalwal K., Shinde V.M., Singh B., Mahadik K.R. (2010). Hypoglycemic and hypolipidemic effect of *Sida rhombifolia* ssp. retusa in diabetic induced animals. Int. J. Phytomed.

[114] Poojari R., Gupta S., Maru G., Khade B., Bhagwat S. (2009). *Sida rhombifolia* ssp. retusa seed extract inhibits DEN-induced murine hepatic preneoplasia and carbon tetrachloride hepatotoxicity. Asian Pac. J. Cancer Prev..

[115] Thounaojam M.C., Jadeja N., Devkar R.V., Ramachandran A.V. (2010). Antioxidant and free radical scavenging activity of *Sida rhomboidea* Roxb methanolic extract determined using different in vitro models. Bol. Latinoam. Caribe Plantas Med. Aromát.

[116] Venkatesh S., Reddy Y.S., Suresh B., Reddy B.M., Ramesh M. (1999). Antinociceptive and anti-inflammatory activity of *Sida rhomboidea* leaves. J. Ethnopharmacol..

[117] Marques A.E.F., Monteiro T.M., dos Santos C.R.B., Piuzevan M.R., de Souza M.d.F.V., Mororó G.T., Alves M.M.d.M., Carvalho F.A.d.A., Arcanjo D.D.R., Gonçalves J.C.R. (2021). Flavonoids isolated from *Sida santaremnensis* H. Monteiro (“Guanxuma”) and evaluation of biological activities. Ciênc. E Nat..

[118] Sangreskopp M.A., Kulkarni P., Mannasaheb B.A. (2013). Antipyretic and antimicrobial potential of *Sida spinosa* Linn. aqueous root extract in rats. Int. J. Phytopharm..

[119] Selvadurai S., Senthamarai R., Sri-Vijaya-Kirubha T., Vasuki K. (2011). Antimicrobial activity of ethanolic extract of the whole plant of *Sida spinosa* Linn. (Malvaceae). J. Nat. Prod. Plant Resour..

[120] Navaneethakrishnan S., Suresh K., Satyanarayana T., Mohideen S., Kiran-Kumar G. (2011). Antimicrobial activity of ethanolic leaf extract of *Sida spinosa* Linn. (Malvaceae). Asian J. Plant Sci. Res..

[121] Tirkey R. (2019). Screening for phytochemical, flavonoid content, antioxidant and anthelmintic potential of *Sida spinosa*. IJPSR.

[122] Silva da Rosa H., Santos M.C., Costa M.T., Salgueiro A., Duarte-da Silva M., Nogueira-Librelotto D.R., Jesse C., Machado M.M., Souza-de Oliveira L.F., Folmer V. (2022). *Sida tuberculata*: In vitro cytotoxicity and in vivo anti-inflammatory effect. J. Ethnopharmacol..

[123] Yamada E.F., Dos Santos-Stein C., Moresco R.N., Bobinski F., Palandi J., Fernandes P.F., Folmer V., da Silva M.D. (2022). Photobiomodulation and *Sida tuberculata* combination declines the inflammation’s markers in knee-induced osteoarthritis. Lasers Med. Sci..

[124] Yamada E.F., Olin L.C., Pontel C.L., da Rosa H.S., Folmer V., da Silva M.D. (2020). *Sida tuberculata* reduces oxidative stress and pain caused by the knee osteoarthritis. J. Ethnopharmacol..

[125] Priya M., Arumugam K., Chakaravarthy C., Chandran K., Bahadur-Sultan A., Zochedh A. (2024). Graph theory network, molecular docking, quantum chemicals and pharmacokinetics-based investigation on phytochemicals from *Sida rhombifolia* against Alzheimer’s disease. PACs.

[126] Rahate S.P., Singh M., Verma A.K., Kumar N., Tiwari N., Shanker K. (2024). Densitometric method for assessment of six specialized metabolites in four *Sida* sp. and its congener *Abutilon indicum*: Targeted metabolomics, greenness assessment, and chemometrics analysis. J. Pharm. Biomed. Anal..

[127] Sharma D., Saluja M.S., Mishra R. (2023). A brief overview of the ethnomedical, pharmacological, and phytochemical uses of *Sida spinosa*. Int. Med. Pharm. Drug Res..

[128] Prakash A., Varma R.K., Ghosal S. (1981). Alkaloid constituents of *Sida acuta*, *S. humilis*, *S. rhombifolia* and *S. spinosa*. Planta Med..

[129] Kumar M., Mani S., Punnoose A.M., Mani E., Mahin R.R.N. (2025). Plant description, phytochemical constituents and pharmacological attributes of the plant *Sida cordata*: A Perspective Review. J. Nat. Remedies.

[130] Khandelwal P., Wadhwani B.D., Rao R.S., Mali D., Vyas P., Kumar T., Nair R. (2024). Exploring the pharmacological and chemical aspects of pyrrolo-quinazoline derivatives in *Adhatoda vasica*. Heliyon.

[131] Sutradhar R.K., Rahman A.M., Ahmad M., Bachar S.C., Saha A., Kumar-Guha S. (2006). Bioactive alkaloid from *Sida cordifolia* Linn. with analgesic and anti-inflammatory activities. IJPT.

[132] Subramanya M.D., Pai S.R., Ankad G.M., Hegde H.V., Roy S., Hoti S.L. (2016). Simultaneous determination of vasicine and vasicinone by High-performance liquid chromatography in roots of eight *Sida* species. AYU.

[133] Dey T., Dutta P., Manna P., Kalita J., Boruah H.P.D., Buragohain A.K., Unni B. (2018). Anti-proliferative activities of vasicinone on lung carcinoma cells mediated via activation of both mitochondria-dependent and independent pathways. Biomol. Ther..

[134] Gad M.Z., Azab S.S., Khattab A.R., Farag M.A. (2021). Over a century since ephedrine discovery: An updated revisit to its pharmacological aspects, functionality and toxicity in comparison to its herbal extracts. Food Funct..

[135] Karmvir-Jat R.K. (2022). Extraction, isolation and standardization of herbal species *Sida*. J. Drug Deliv. Ther..

[136] Imran M., Patil S.P., Abourehab M.A.S., Aeri V., Kesharwani P. (2022). Quality by design-based optimization of Soxhlet extraction and identification of ephedrine by a HPTLC method for *Sida rhombifolia* and *Sida Cordifolia*. Biomed. Chromatogr..

[137] Selvadurai S., Senthamarai R., Kiruba T., Nagarajan G., Gayasuddin M. (2012). Antidiabetic activity of whole plant of *Sida spinosa* Linn. (Malvaceae) on diabetic induced rats. IJRPP.

[138] Głowacka K., Wiela-Hojeńska A. (2021). Pseudoephedrine-Benefits and Risks. Int. J. Mol. Sci..

[139] Tanumihadja M., Mattulada I.K., Natsir N., Subehan S., Mandey F., Muslimin L. (2019). Structural assessment of chemical constituent of Sidaguri (*Sida rhombifolia* Linn) and its ability to inhibit cyclooxygenase. Pesqui. Bras. Odontopediatria Clin. Integr..

[140] Sun H., Zhu X., Cai W., Qiu L. (2017). Hypaphorine attenuates lipopolysaccharide-induced endothelial inflammation via regulation of TLR4 and PPAR-γ dependent on PI3K/Akt/mTOR signal pathway. Int. J. Mol. Sci..

[141] Joseph B., Ajisha A.U., Kumari S., Sujatha S. (2011). Effect of bioactive compounds and its pharmaceutical activities of *Sida cordifolia* (Linn.). Int. J. Biol. Med. Res..

[142] Selvadurai S., Shanmugapandiyan P. (2022). Preliminary phytochemical analysis on the leaves extracts of *Sida acuta* Burm.f. and *Sida rhombifolia* Linn. family Malvaceae. RJPT.

[143] Zhou D., Wei H., Jiang Z., Li X., Jiao K., Jia X., Hou Y., Li N. (2017). Natural potential neuroinflammatory inhibitors from *Alhagi sparsifolia* Shap. Bioorg Med. Chem. Lett..

[144] da Silva R.G., Almeida T.C., Reis A.C.C., Filho S.A.V., Brandão G.C., da Silva G.N., de Sousa H.C., de Almeida V.L., Lopes J.C.D., de Souza G.H.B. (2021). In silico pharmacological prediction and cytotoxicity of flavonoids glycosides identified by UPLC-DAD-ESI-MS/MS in extracts of *Humulus lupulus* leaves cultivated in Brazil. Nat. Prod. Res..

[145] Agraharam G., Girigoswami A., Girigoswami K. (2022). Myricetin: A Multifunctional Flavonol in Biomedicine. Curr. Pharmacol. Rep..

[146] Kiziltaş H. (2022). Comprehensive evaluation of *Reseda lutea* L. (Wild Mignonette) and 7 isolated flavonol glycosides: Determination of antioxidant activity, anti-Alzheimer, antidiabetic and cytotoxic effects with in vitro and in silico methods. Turk. J. Chem..

[147] Prata M.N.L., Charlie-Silva I., Gomes J.M.M., Barra A., Berg B.B., Paiva I.R., Melo D.C., Klein A., Romero M.G.M.C., Oliveira C.C. (2020). Anti-inflammatory and immune properties of the peltatoside, isolated from the leaves of *Annona crassiflora* Mart., in a new experimental model zebrafish. Fish. Shellfish. Immunol..

[148] Ghorbani A. (2017). Mechanisms of antidiabetic effects of flavonoid rutin. Biomed. Pharmacother..

[149] Cristina da Costa-Araldi I., Piber-de Souza T., de Souza-Vencato M., de Andrade-Fortes T., Emanuelli-Mello C.B., Sorraila-de Oliveira J., Dornelles G.L., Melazzo-de Andrade C., Maciel R.M., Danesi C.C. (2021). Preclinical safety assessment of the crude extract from *Sida rhombifolia* L. aerial parts in experimental models of acute and repeated-dose 28 days toxicity in rats. Regul. Toxicol. Pharmacol..

[150] Muneeswari P., Kunnathupara-Bhaskaran S., Poornima K. (2019). Identification of active pharmaceuticals of *Sida acuta* Burm. f. leaves using GC-MS and HPTLC fingerprinting. IJPSR.

[151] Alrumaihi F., Almatroodi S.A., Alharbi H.O.A., Alwanian W.M., Alharbi F.A., Almatroudi A., Rahmani A.H. (2024). Pharmacological potential of kaempferol, a flavonoid in the management of pathogenesis via modulation of inflammation and other biological activities. Molecules.

[152] Li X., Tian Y., Wang T., Lin Q., Feng X., Jiang Q., Liu Y., Chen D. (2017). Role of the p-Coumaroyl Moiety in the Antioxidant and Cytoprotective Effects of Flavonoid Glycosides: Comparison of Astragalin and Tiliroside. Molecules.

[153] Padhy I., Dash P., Sahu R.K., Saha G. (2019). Anti-inflammatory activity of isolated flavonoid from *Sida cordifolia* leaves extract. Int. J. Med. Res. Health Sci..

[154] Gong G., Guan Y.Y., Zhang Z.L., Rahman K., Wang S.J., Zhou S., Luan X., Zhang H. (2020). Isorhamnetin: A review of pharmacological effects. Biomed. Pharmacother..

[155] Esmeeta A., Adhikary S., Dharshnaa V., Swarnamughi P., Ummul-Maqsummiya Z., Banerjee A., Pathak S., Duttaroy A.K. (2022). Plant-derived bioactive compounds in colon cancer treatment: An updated review. Biomed. Pharmacother..

[156] Maiyo F., Moodley R., Singh M. (2016). Phytochemistry, cytotoxicity and apoptosis studies of β-sitosterol-3-O-glucoside and β-amyrin from *Prunus africana*. Afr. J. Tradit. Complement. Altern. Med..

[157] Pooja J., Vivek K., Deepti T., Hritika G., Aman A., Vijai M. (2024). Phytochemical composition of *Sida rhombifolia* ssp. retusa (L.) Bross.: A comprehensive GC/MS analysis. Xi’an Shiyou Daxue Xuebao (Ziran Kexue Ban).

[158] Ganesh M., Mohankumar M. (2017). Extraction and identification of bioactive components in *Sida cordata* (Burm.f.) using gas chromatography-mass spectrometry. J. Food Sci. Technol..

[159] Islam M.T., Ali E.S., Uddin S.J., Shaw S., Islam M.A., Ahmed M.I., Chandra-Shill M., Karmakar U.K., Yarla N.S., Khan I.N. (2018). Phytol: A review of biomedical activities. Food Chem. Toxicol..

[160] Huang Z.R., Lin Y.K., Fang J.Y. (2009). Biological and pharmacological activities of squalene and related compounds: Potential uses in cosmetic dermatology. Molecules.

[161] Moeurng S., Posridee K., Kamkaew A., Thaiudom S., Oonsivilai A., Oonsivilai R. (2024). Identification of Pheophytin a and Hydroxy Pheophytin a from Rang Chuet (*Thunbergia laurifolia* Linn.) as Potent NQO-1 Inducers in Liver Cells. Foods.

[162] Chowdhury A., Ashraful-Alam M., Rahman M., Rashid M. (2008). Phytochemical and Biological Investigations of *Sida rhomboidea* Linn. IJAM.

[163] El Omari N., Jaouadi I., Lahyaoui M., Benali T., Taha D., Bakrim S., El Menyiy N., El Kamari F., Zengin G., Bangar S.P. (2022). Natural sources, pharmacological properties, and health benefits of daucosterol: Versatility of actions. Appl. Sci..

[164] Si W., Chen Z., Bei J., Chang S., Zheng Y., Gao L., Zhao G., Li X., Zhang D. (2024). Stigmasterol alleviates neuropathic pain by reducing Schwann cell-macrophage cascade in DRG by modulating IL-34/CSF1R. CNS Neurosci. Ther..

[165] Banni M., Jayaraj M. (2023). Identification of bioactive compounds of leaf extracts of *Sida cordata* (Burm.f.) Borss. Waalk. by GC/MS analysis. Appl. Biochem. Biotechnol..

[166] Yadav S., Aggarwal P., Khan F., Khodve G., Padhy D.S., Yadav P., Banerjee S. (2023). β-sitosterol protects against aluminium chloride-mediated neurotoxicity. Curr. Alzheimer Res..

[167] Tripathi N., Kumar S., Singh R., Singh C.J., Singh P., Varshney V. (2013). Isolation and identification of gamma-sitosterol by GC-MS from roots of *Girardinia heterophylla*. Orient. J. Chem..

[168] da Rosa H.S., Salgueiro A.C., Colpo A.Z., Paula F.R., Mendez A.S., Folmer V. (2016). *Sida tuberculata* (Malvaceae): A study based on development of extractive system and in silico and in vitro properties. Braz. J. Med. Biol. Res..

[169] Gong X., Bai M., Lu G., Yang B., Lei T., Shizhi J. (2022). Total synthesis of murraol, (E)-Suberenol and toward the collective total synthesis of exotines A, cnidimonins A-Cetc. Tetrahedron.

[170] Tavakoli S., Delnavazi M.R., Hadjiaghaee R., Jafari-Nodooshan S., Khalighi-Sigaroodi F., Akhbari M., Hadjiakhoondi A., Yassa N. (2018). Bioactive coumarins from the roots and fruits of *Ferulago trifida* Boiss., an endemic species to Iran. Nat. Prod. Res..

[171] Parama D., Girisa S., Khatoon E., Kumar A., Alqahtani M.S., Abbas M., Sethi G., Kunnumakkara A.B. (2022). An overview of the pharmacological activities of scopoletin against different chronic diseases. Pharmacol. Res..

[172] Nagarajan S., Murali R. (2022). An overview of the traditional importance, phytochemistry, and pharmacological properties of *Sida acuta* Burm.f. Ann. Phytomed.

[173] Amine H., Benomar Y., Taouis M. (2021). Publisher Correction: Palmitic acid promotes resistin induced insulin resistance and inflammation in SH SY5Y human neuroblastoma. Sci. Rep..

[174] Santa-María C., López-Enríquez S., Montserrat-de la Paz S., Geniz I., Reyes-Quiroz M.E., Moreno M., Palomares F., Sobrino F., Alba G. (2023). Update on anti-inflammatory molecular mechanisms induced by Oleic Acid. Nutrients.

[175] Nava-Lauson C.B., Tiberti S., Corsetto P.A., Conte F., Tyagi P., Machwirth M., Ebert S., Loffreda A., Scheller L., Sheta D. (2023). Linoleic acid potentiates CD8+ T cell metabolic fitness and antitumor immunity. Cell Metab..

[176] Zhang Y., Wu Q., Zhang L., Wang Q., Yang Z., Liu J., Feng L. (2019). Caffeic acid reduces A53T α-synuclein by activating JNK/Bcl-2-mediated autophagy in vitro and improves behaviour and protects dopaminergic neurons in a mouse model of Parkinson’s disease. Pharmacol. Res..

[177] Kurniawan Y.S., Priyangga K.T.A., Jumina, Pranowo H.D., Sholikhah E.N., Zulkarnain A.K., Fatimi H.A., Julianus J. (2021). An update on the anticancer activity of xanthone derivatives: A review. Pharmaceuticals.

[178] Zeng J., Xie Z., Chen L., Peng X., Luan F., Hu J., Xie H., Liu R., Zeng N. (2024). Rosmarinic acid alleviate CORT-induced depressive-like behavior by promoting neurogenesis and regulating BDNF/TrkB/PI3K signaling axis. Biomed. Pharmacother..

[179] Paiva L., Goldbeck R., dos Santos-Dantas W., Squina F. (2013). Ferulic acid and derivatives: Molecules with potential application in the pharmaceutical field. Braz. J. Pharm. Sci..

[180] Chumpol W., Tavichakorntrakool R., Lulitanond A., Daduang J., Saisud P., Sribenjalux P., Prasongwatana V., Boonsiri P. (2018). The antibacterial activity of the aqueous extract of *Sida acuta* Burm. F. Southeast. Asian J. Trop. Med. Public. Health.

[181] Sreedevi C.D., Latha P.G., Ancy P., Suja S.R., Shyamal S., Shine V.J., Sini S., Anuja G.I., Rajasekharan S. (2009). Hepatoprotective studies on *Sida acuta* Burm. f. J. Ethnopharmacol..

[182] Ezeako E.C., Nworah F.N., Osuji D.O. (2023). Phytocompounds, antioxidant potential, and inhibitory actions of ethanolic leaf fraction of *Sida linifolia* Linn. (Malvaceae) on enzymes linked to infammation, diabetes, and neurological disorders. Futur. J. Pharm. Sci..

[183] Bai J., Zhang Y., Tang C., Hou Y., Ai X., Chen X., Zhang Y., Wang X., Meng X. (2021). Gallic acid: Pharmacological activities and molecular mechanisms involved in inflammation-related diseases. Biomed. Pharmacother..

[184] Chandrasekara A., Shahidi F. (2018). Herbal beverages: Bioactive compounds and their role in disease risk reduction—A review. J. Tradit. Complement. Med..

[185] Breuss J.M., Atanasov A.G., Uhrin P. (2019). Resveratrol and Its Effects on the Vascular System. Int. J. Mol. Sci..

[186] Pubmed https://pubmed.ncbi.nlm.nih.gov/?term=Sida+rhombifolia&size=50.

[187] Hui Y., Wang X., Yu Z., Fan X., Cui B., Zhao T., Mao L., Feng H., Lin L., Yu Q. (2020). Scoparone as a therapeutic drug in liver diseases: Pharmacology, pharmacokinetics and molecular mechanisms of action. Pharmacol. Res..

[188] Sun Y., Zhu D., Kong L., Du W., Qu L., Yang Y., Rao G., Huang F., Tong X. (2024). Vasicine attenuates atherosclerosis via lipid regulation, inflammation inhibition, and autophagy activation in ApoE-/- mice. Int. Immunopharmacol..

